# Ribosomal Multi-Operon Diversity: An Original Perspective on the Genus *Aeromonas*

**DOI:** 10.1371/journal.pone.0046268

**Published:** 2012-09-27

**Authors:** Frédéric Roger, Brigitte Lamy, Estelle Jumas-Bilak, Angeli Kodjo, the colBVH study group, Carmagnol F., Chachaty E., Alba-Sauviat C., Auvray C., Barraud D., Benseddik Z., Bertrou A., Bessis F., Biessy H., Blanc V., Boucaud-Maitre Y., Brunet P., Michel A., Cancet B., Carrere J., Cecille A., Chambreuil G., Chantelat P., Chardon H., Charrel C., De Montclos H., Decousser J.W., Delarbre J. M., Gravet A., Deligne D., Denoix C., Deregnaucourt J., Desroys du Roure F., Dubourdieu S., El Harrif Z., Eloy C., Evers A., Febvre C., Fevre D., Gabriel S., Galanti M. J., Garnotel E., Gavignet M., Geffroy F., Grise G., Gros I., Hermes I., Heurte J., Heusse E., Jan D., Jaouen E., Laluque S., Lamarca R., E. Laurens, Le Coustumier A., Lecaillon E., Lemble C., Leneveu M., Leotard S., Letouzey M. N., Malbrunot C., Menouni O., Morel M., Olive C., Pangon B., Paul J. G., Perez J. M., Pouedras P., Pressac D., Sanchez R., Scat Y., Secher A., Semon J., Simeon D., Simonin C., Thellier J. P., Tourand B., Vachée A., Varache C., Vaucel J., Vautrin A. C., Verhaeghe A., Villemain M., Villeneuve L., Hélène Marchandin

**Affiliations:** Centre Hospitalier de Cannes; Institut Gustave Roussy; Centre Hospitalier de Chaumont; Centre Hospitalier de Charleville-Mézières; Centre Hospitalier de Gonesse; Centre Hospitalier de Chartres; Centre Hospitalier de Carcassone; Centre Hospitalier de Cherbourg; Centre Hospitalier de La Rochelle; Centre Hospitalier d’Antibes-Juan-les-pins; Centre Hospitalier Saint Joseph-Saint Luc, Lyon; Hôpital Saint Joseph, Marseille; Centre Hospitalier de Villeneuve/Lot; Centre Hospitalier de Hyères; Centre Hospitalier de Digne-les-bains; Centre Hospitalier de La Roche/Yon; Centre Hospitalier de Vesoul; Centre Hospitalier d’Aix-en-Provence; Centre Hospitalier de Salon de Provence; Centre Hospitalier de Bourg-en-Bresse; Hôpital Antoine Beclère, Paris; Centre Hospitalier de Mulhouse; Centre Hospitalier de Remiremont; Centre Hospitalier Universitaire de La Réunion; Hôpital Léopold Bellan, Paris; Centre Hospitalier de Chatellerault; Centre Hospitalier de Gisors; Centre Hospitalier de Libourne; Centre Hospitalier de Troyes; Centre Hospitalier de Annonay; Centre Hospitalier de Montbéliard; Centre Hospitalier de Vienne; Centre Hospitalier Princesse Grace, Monaco; Centre Hospitalier de Coulommiers; Hôpital d’Instruction des Armées Lavean à Marseille; Centre Hospitalier de Lavaur; Centre Hospitalier de Quimper; Centre Hospitalier de Elbeuf-Louviers; Centre Hospitalier de St Denis; Centre Hospitalier de St-Malo; Centre Hospitalier de Beauvais; Centre Hospitalier de Bayeux; Centre Hospitalier de Laval; Centre Hospitalier de Sablé/Sarthe; Centre Hospitalier de Montluçon; Centre Hospitalier de Narbonne; Centre Hospitalier de Belfort; Centre Hospitalier de Cahors; Centre Hospitalier de Perpignan; Centre Hospitalier de Selestat; Centre Hospitalier Intercommunal de Poissy Saint-Germain-en-Laye; Centre Hospitalier de Grasse; Centre Hospitalier de Villefranche/saone; Centre Hospitalier de Corbeil-Essonnes; Centre Hospitalier de Montceau-les-Mines; Centre Hospitalier de Le Havre; Centre Hospitalier Universitaire de Fort de France; Centre Hospitalier de Versailles; Centre Hospitalier de Boulogne/mer; Centre Hospitalier Universitaire de Pointe-à-Pitre; Centre Hospitalier de Vannes; Centre Hospitalier de Tulle; Centre Hospitalier de Périgueux; Centre Hospitalier de Pontivy; Centre Hospitalier de Dreux; Centre Hospitalier de Chalon-sur-Saone; Centre Hospitalier de Langres; Centre Hospitalier de Macon; Centre Hospitalier de Château-Thierry; Centre Hospitalier d’Alès; Centre Hospitalier de Roubaix; Centre Hospitalier du Mans; Centre Hospitalier de Saint-Brieux; Centre Hospitalier Universitaire de Saint-Etienne; Centre Hospitalier de Dunkerke; Centre Hospitalier d’Aurillac; Centre Hospitalier d’Aubagne; 1 UMR 5119 ECOSYM, Equipe Pathogènes et Environnements, U.F.R. des Sciences Pharmaceutiques et Biologiques, Université Montpellier 1, Montpellier, France; 2 Laboratoire de Bactériologie, Hôpital Arnaud de Villeneuve, Centre Hospitalier Régional Universitaire de Montpellier, Montpellier, France; 3 Groupe d’Etude Français des *Aeromonas* (GFA), Lyon, France; 4 Laboratoire d’Hygiène hospitalière, Centre Hospitalier Régional Universitaire de Montpellier, Montpellier, France; 5 CNRS UMR 5557 Ecologie microbienne, VetAgro Sup Campus vétérinaire de Lyon, Université Claude Bernard Lyon 1, Marcy-l’Étoile, France; 6 ColBVH, Collège de bactériologie, virologie et hygiène des hôpitaux généraux, le Chesnay, France; Universidad Miguel Hernandez, Spain

## Abstract

16S rRNA gene (*rrs*) is considered of low taxonomic interest in the genus *Aeromonas*. Here, 195 *Aeromonas* strains belonging to populations structured by multilocus phylogeny were studied using an original approach that considered Ribosomal Multi-Operon Diversity. This approach associated pulsed-field gel electrophoresis (PFGE) to assess *rrn* operon number and distribution across the chromosome and PCR-temporal temperature gel electrophoresis (TTGE) to assess *rrs* V3 region heterogeneity. Aeromonads harbored 8 to 11 *rrn* operons, 10 operons being observed in more than 92% of the strains. Intraspecific variability was low or nul except for *A. salmonicida* and *A. aquariorum* suggesting that large chromosomic rearrangements might occur in these two species while being extremely rarely encountered in the evolution of other taxa. *rrn* operon number at 8 as well as PFGE patterns were shown valuable for taxonomic purpose allowing resolution of species complexes. PCR-TTGE revealed a high rate of strains (41.5%) displaying intragenomic *rrs* heterogeneity. Strains isolated from human samples more frequently displayed intragenomic heterogeneity than strains recovered from non-human and environmental specimens. Intraspecific variability ranged from 0 to 76.5% of the strains. The observation of species-specific TTGE bands, the recovery of identical V3 regions in different species and the variability of intragenomic heterogeneity (1–13 divergent nucleotides) supported the occurrence of mutations and horizontal transfer in aeromonad *rrs* evolution. Altogether, the presence of a high number of *rrn* operon, the high proportion of strains harboring divergent *rrs* V3 region and the previously demonstrated high level of genetic diversity argued in favor of highly adaptative capabilities of aeromonads. Outstanding features observed for *A. caviae* supported the ongoing process of adaptation to a specialized niche represented by the gut, previously hypothesized. 16S rRNA gene is an informative marker in the genus *Aeromonas* for both evolutionary and polyphasic taxonomic studies provided that multi-operon fingerprinting approaches are used.

## Introduction

The genus *Aeromonas* groups waterborne gram-negative bacilli considered as opportunistic pathogens for a wide range of animals including humans. Currently, 25 species are recognized in the genus. 16S rRNA (*rrs*) gene is considered as a non-informative marker in the genus because most species shared more than 99% of their nucleotide positions [1]–[4]. Considering this low polymorphism, several attempts have been made to identify species by 16S rRNA gene-restriction fragment length polymorphism (RFLP) [5], [6] but atypical patterns [7], [8] that were later explained by sequence heterogeneities among 16S rRNA gene copies in a same genome [9], [10] impaired this approach. Due to these limits, 16S rRNA gene sequence was subsequently scarcely studied in the genus *Aeromonas* and multilocus sequence analysis is now the reference approach to structure populations and describe new species in the genus [2]–[4], [11]–[13].

Beside the sequence polymorphism, ribosomal operons display diversity in terms of copy number and copy heterogeneity in bacterial genomes [14], [15]. The genus *Aeromonas* is a valuable example that illustrates such diversity. Eight to ten copies of the 16S rRNA genes have been reported in totally sequenced *Aeromonas* genomes [16]–[19] and significant intragenomic microheterogeneities have been suggested using different approaches [9], [10], [20].

Here we evaluated the “Ribosomal Multi-Operon Diversity” (RiMOD), i.e., the diversity of the chromosomal *rrn* copy organisation and heterogenity, towards taxonomy and evolution of aeromonads. For this purpose, we used pulsed-field gel electrophoresis to determine *rrn* operon number and distribution across the chromosome and PCR-temporal temperature gel electrophoresis (TTGE) to assess *rrs* heterogeneity in a population of *Aeromonas* field isolates and reference strains from diverse origins previously structured by multilocus phylogenetic analysis (MLPA) [13]. We reported the features of *rrs* heterogeneity at the intrapopulation, intraspecific and intragenomic levels, and the variability of *rrn* number and chromosomal distribution at both inter- and intra-specific levels. Our approach showed that ribosomal diversity when studied at the multi-operon family level is an informative marker regarding evolution and taxonomy in the genus *Aeromonas*. In addition, we showed that RiMOD study results supported the structure in species complexes previously suggested for the genus *Aeromonas* on the basis of other genetic and genomic traits [13] and more generally related to “generalist” bacterial pathogens with sympatric lifestyles [21].

## Materials and Methods

### Bacterial Strains

Strain collection was as described in Roger *et al*. [13]. Briefly, a total of 195 strains of *Aeromonas* spp., including 62 type and reference strains were analyzed. Origin distribution of these strains reflected the different bacterial lifestyles in the genus with 115, 39 and 41 isolates recovered from human clinical samples, non-human animal samples and environmental samples, respectively. The strain collection included type strains of 25 species and a representative strain of hybridization group (HG) 11. Using MLPA, the population was distributed in eleven species corresponding to *Aeromonas allosaccharophila* (n = 3), *Aeromonas aquariorum* (n = 8), *Aeromonas caviae* (n = 34), *Aeromonas hydrophila* (n = 35), *Aeromonas jandaei* (n = 2), *Aeromonas media* (n = 6), *Aeromonas piscicola* (n = 3), *Aeromonas salmonicida* (n = 8), *Aeromonas sobria* (n = 5), *Aeromonas tecta* (n = 3), *Aeromonas veronii* (n = 71). Fourteen species were represented by their type strain only: *Aeromonas bestiarum*, *Aeromonas bivalvium*, *Aeromonas diversa*, *Aeromonas encheleia*, *Aeromonas enteropelogenes*/*trota*, *Aeromonas eucrenophila*, *Aeromonas fluvialis*, *Aeromonas molluscorum*, *Aeromonas popoffii*, *Aeromonas sanarellii*, *Aeromonas simiae*, *Aeromonas schubertii*, *Aeromonas taiwanensis* and *Aeromonas rivuli*. Finally, the strain CCM 1271 was representative of an unknown taxon (Table S1).

### In silico Analysis

Complete genome sequences of strains *A. hydrophila* subsp. *hydrophila* ATCC 7966^T^ (GenBank accession number NC_008570), *A. salmonicida* subsp. *salmonicida* A449 (NC_009348), and *A. veronii* B565 (NC_015424) were analyzed *in silico* for *rrs* copy number, *rrs* position determination and *rrs* sequence heterogeneity using Biological sequence aligment editor (BioEdit) programme version 7.0.9 [22] (http://www.mbio.ncsu.edu/bioedit/bioedit.html).

### PFGE

Genomic DNA was prepared in agarose plugs as previously described by Miranda *et al*. [23] and adapted by Roger *et al*. [13]. DNA was digested at 37°C with 1 U of the intronic endonuclease I-*Ceu*I (New England BioLabs, Hertfordshire, United Kingdom) according to the manufacturer’s recommendations. I-*Ceu*I fragments were resolved using a CHEF-DRII apparatus (Bio-Rad Laboratories, Hercules, CA) in a 0.8% agarose gel in 0.5X Tris-Borate-EDTA (TBE) buffer added with 50 µM of thiourea at 4.5 V/cm and at 10°C. Pulse ramps were 60 to 10 s for 35 h. The gel was stained with ethidium bromide and photographed under UV light. Number of *rrn* copies was deduced from the number of I-*Ceu*I-generated fragments. Sizes determined in this study were measured by comparison with lambda concatemer (New England BioLabs) used as size standard.

### PCR-TTGE

Amplification by PCR of a 199 bp-fragment overlapping the 16S rDNA variable region V3 and TTGE were performed as described previously [24]. Bands were cut out of the gel, DNA was amplified using HDA1 and HDA2 primers as previously described and PCR products were sequenced on an Applied Biosystems automatic sequencer (Cogenics) by using the forward primer HDA1 [24]. Bands for which no interpretable sequences were obtained were subjected to a second run of PCR-TTGE followed by a re-sequencing as described above. TTGE bands were visually detected and analyzed by migration distance measuring after standardization. Each band with distinct distance of migration was designated by a number and TTGE profiles were indicated by a combination of band numbers separated by a + sign. A specifically designed ladder corresponding to a mix of V3 region amplification products from different strains showing PCR-TTGE pattern 1+4+15+30 was loaded on all the TTGE gels. The presence of more than one TTGE band, i.e., 16S rRNA gene V3 region copies with distinct sequences, in an isolate will be referred to hereafter as harboring intragenomic heterogeneity, while the presence of different TTGE bands in different isolates of a species will be referred to as intraspecific heterogeneity.

### Statistical Analyses

Qualitative variables were compared with the Chi-square test with the Bonferroni’s correction where required; a *P* value ≤0.05 was considered to reflect significance. All computations were done with R project software (http://www.r-project.org).

## Results

### *rrs* Sequence Diversity and Heterogeneity

The 195 strains of the collection were studied by 16S rRNA gene PCR-TTGE. Representative patterns are shown in Figure S1. Fifty-seven PCR-TTGE patterns associating bands with 34 different migration distances were observed in the population (Table 1, Table S1). Multiband TTGE patterns, revealing *rrs* heterogeneity, were more frequently observed (n = 45, 78.9%) than patterns composed by a unique band (n = 12, 21.1%). Among the 195 strains, 81 (41.5%) showed heterogeneity between their *rrs* copies. Up to four bands were combined within a pattern; however, the 2-band patterns were the most frequently observed (n = 27, 60% of the 45 heterogeneous patterns). Patterns with 3 or 4 different bands (13 and 5 patterns, respectively) were less frequently observed. Heterogeneity frequency differed significantly between the groups of isolates recovered from human, animal and environmental samples (*P* value = 0.01), V3 *rrs* heterogeneity being observed in 51.3% of isolates from human origin (59 of the 115 strains) compared to 28.2% of non-human animal isolates (11 of the 39 strains) and 26.8% of environmental isolates (11 of the 41 strains). Difference in heterogeneity frequency between strains purchased from collections and field isolates was not significant (*P* value = 0.078).

**Table 1:** Occurrence and distribution of the 34 PCR-TTGE bands observed among the *Aeromonas* population.

PCR-TTGE band^a^	Occurrence in the population/number of taxa with band occurence (n/n)	Number of patterns with band^b^	Occurence of band according to the taxon (n)^c^																											
			*A. veronii* (n = 71)	*A. hydrophila* (n = 35)	*A. caviae* (n = 34)	*A. aquariorum* (n = 8)	*A. salmonicida* (n = 8)	*A. media* (n = 6)	*A. sobria* (n = 5)	*A. allosaccharophila* (n = 3)	*A. piscicola* (n = 3)	*A. tecta* (n = 3)	*A. jandaei* n = (2)	*A. bestarium*	*A. bivalvium*	*A. diversa*	*A. encheleia*	*A. enteropelogenes*	*A. eucrenophila*	*A. fluvialis*	*A. molluscorum*	*A. popoffii*	*A. sanarellii*	*A. rivuli*	*A. simiae*	*A. schubertii*	*A. taiwanensis*	*A. trota*	HG11	Strain CCM 1271
**1**	42/4	15	–	**35**	5	–	–	–	–	–	–	–	–	–	–	–	1	–	–	–	1	–	–	–	–	–	–	–	–	–
**2**	6/4	**2**	–	3	–	1	–	1	–	–	–	–	–	–	–	–	–	–	–	–	1	–	–	–	–	–	–	–	–	–
**3**	32/6	14	2	–	19	**8**	–	–	–	–	–	–	–	–	–	–	–	1	–	–	–	–	1	–	–	–	1	–	–	–
**4**	47/7	18	7	1	**34**	1	–	–	–	–	–	–	**2**	–	–	–	–	1	–	–	–	–	–	–	–	–	–	1	–	–
**5**	3/1	**1**	–	***3***	–	–	–	–	–	–	–	–	–	–	–	–	–	–	–	–	–	–	–	–	–	–	–	–	–	–
**6**	1/1	**1**	–	–	***1***	–	–	–	–	–	–	–	–	–	–	–	–	–	–	–	–	–	–	–	–	–	–	–	–	–
**8**	2/2	**2**	–	1	1	–	–	–	–	–	–	–	–	–	–	–	–	–	–	–	–	–	–	–	–	–	–	–	–	–
**9**	1/1	**1**	–	***1***	–	–	–	–	–	–	–	–	–	–	–	–	–	–	–	–	–	–	–	–	–	–	–	–	–	–
**10**	2/1	**2**	–	***2***	–	–	–	–	–	–	–	–	–	–	–	–	–	–	–	–	–	–	–	–	–	–	–	–	–	–
**11**	78/5	11	70	–	5	–	–	1	–	–	–	–	–	–	–	1	–	–	–	–	–	–	–	–	1	–	–	–	–	–
**12**	2/1	**2**	–	–	***2***	–	–	–	–	–	–	–	–	–	–	–	–	–	–	–	–	–	–	–	–	–	–	–	–	–
**13**	4/2	1	1	–	–	–	–	–	–	**3**	–	–	–	–	–	–	–	–	–	–	–	–	–	–	–	–	–	–	–	–
**14**	1/1	**1**	–	–	–	–	–	–	–	–	–	–	–	–	–	–	–	–	–	***1***	–		–	–	–	–	–	–	–	–
**15**	21/8	10	8	–	3	–	–	3	–	–	–	**3**	–	–	–	–	–	–	1	–	–	1	–	–	–	–	–	1	1	–
**16**	1/1	**1**	***1***	–	–	–	–	–	–	–	–	–	–	–	–	–	–	–	–	–	–	–	–	–	–	–	–	–	–	–
17	3/1	**1**	***3***	–	–	–	–	–	–	–	–	–	–	–	–	–	–	–	–	–	–	–	–	–	–	–	–	–	–	–
**18**	10/3	**7**	–	1	8	–	–	–	–	–	–	–	–	–	–	–	–	–	–	–	–	–	1	–	–	–	–	–	–	–
**19**	1/1	**1**	–	–	***1***	–	–	–	–	–	–	–	–	–	–	–	–	–	–	–	–	–	–	–	–	–	–	–	–	–
**20**	3/1	**3**	–	–	–	–	–	***3***	–	–	–	–	–	–	–	–	–	–	–	–	–	–	–	–	–	–	–	–	–	–
**21**	4/1	4	–	–	–	–	–	***4***	–	–	–	–	–	–	–	–	–	–	–	–	–	–	–	–	–	–	–	–	–	–
**24**	1/1	**1**	–	–	–	–	–	–	–	–	–	–	–	–	–	–	–	–	–	–	***1***	–	–	–	–	–	–	–	–	–
25	1/1	**1**	–	–	–	–	–	–	–	–	–	–	–	–	–	–	–	–	–	–	***1***	–	–	–	–	–	–	–	–	–
**27**	1/1	1	–	–	–	–	–	–	–	–	–	–	–	–	***1***	–	–	–	–	–	–	–	–	–	–	–	–	–	–	–
**29**	1/1	**1**	–	–	–	–	–	–	–	–	–	–	–	–	–	–	–	–	–	–	–	***1***	–	–	–	–	–	–	–	–
**30**	20/8	4	–	4	–	–	**8**	–	–	–	**3**	–	–	1	–	–	1	–	–	1	–	–	–	–	–	–	–	–	1	1
**31**	1/1	**1**	–	–	–	–	–	–	–	–	–	–	–	–	–	–	–	–	–	–	–	–	–	–	–	–	***1***	–	–	–
**32**	1/1	1	–	–	–	–	–	–	–	–	–	–	–	–	–	–	–	–	–	–	–	–	–	***1***	–	–	–	–	–	–
33	1/1	**1**	***1***	–	–	–	–	–	–	–	–	–	–	–	–	–	–	–	–	–	–	–	–	–	–	–	–	–	–	–
35	2/2	**2**	–	1	–	1	–	–	–	–	–	–	–	–	–	–	–	–	–	–	–	–	–	–	–	–	–	–	–	–
**36**	1/1	**1**	–	–	–	–	–	–	–	–	–	–	–	–	–	–	–	***1***	–	–	–	–	–	–	–	–	–	–	–	–
**37**	1/1	1	–	–	–	–	–	–	–	–	–	–	–	–	–	–	–	–	–	–	–	–	–	–	–	***1***	–	–	–	–
**38**	5/1	1	–	–	–	–	–	–	***5***	–	–	–	–	–	–	–	–	–	–	–	–	–	–	–	–	–	–	–	–	–
39	1/1	**1**	–	***1***	–	–	–	–	–	–	–	–	–	–	–	–	–	–	–	–	–	–	–	–	–	–	–	–	–	–
**40**	1/1	**1**	–	–	***1***	–	–	–	–	–	–	–	–	–	–	–	–	–	–	–	–	–	–	–	–	–	–	–	–	–
**Number of pattern**			8	11	17	4	1	6	1	1	1	1	1	1	1	1	1	1	1	1	1	1	1	1	1	1	1	1	1	1
**Number of heterogeneous pattern**			6	10	16	3	0	4	0	0	0	0	0	0	0	0	1	1	0	1	1	1	1	0	0	0	1	1	1	0
**Number of strains with ** ***rrs*** ** V3 region heterogeneity**			22	17	26	3	0	4	0	0	0	0	0	0	0	0	1	1	0	1	1	1	1	0	0	0	1	1	1	0

a Bold type indicates bands which were successfully sequenced (GenBank accession numbers JX014439-JX014453 and JX453432-JX453445).

b Bold type indicates that all patterns with the corresponding band showed heterogeneity.

c Bold and italics, bands only found in one taxon; bold and underlined, bands found in all representatives of a taxon for taxa including more than one strain (bold, italics and underlined combined for band 38 in *A. sobria*).

Among the 34 TTGE bands with visually distinct migration distances observed for the overall population, 29 were confirmed as distinct by sequencing. For 15 of these bands, sequences were obtained after the first run of PCR-TTGE (GenBank accession numbers JX014439-JX014453) while for the 14 other bands a second run of PCR-TTGE was required (Genbank accession number JX453432-JX453445). Sequences could not be obtained for the 5 remaining bands because sequencings were either unrealizable due to very faint bands or resulted in uninterpretable bulk sequences despite the second run of PCR-TTGE. These 5 bands were observed in 8 strains belonging to 4 taxa (Table 1). The 49 patterns with full band sequencing displayed heterogeneity between V3 *rrs* copies ranging from 1 to 13 nucleotides (151 nt-alignment) (Table S1). The diversity of bands and profiles surpassed the number of taxa identified by MLPA in the collection, revealing variation of V3 *rrs* content in a same species.

**Figure 1:**
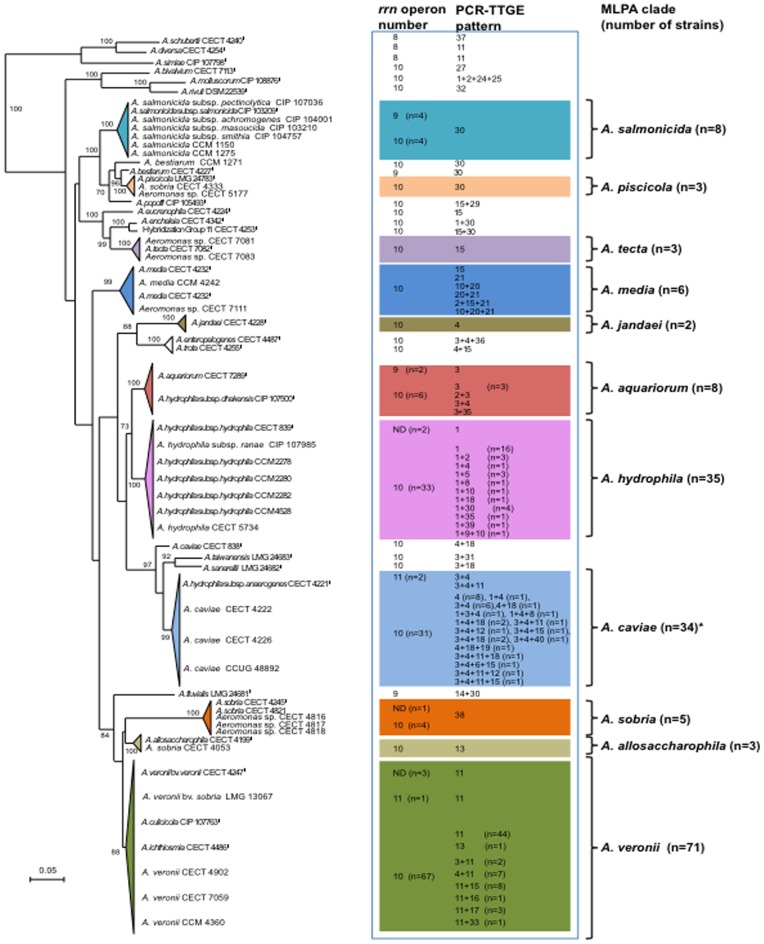
Number of *rrn* operons and PCR-TTGE patterns for the 195 *Aeromonas* strains of the study. Results are presented according to the structure of the population observed in multilocus phylogenetic analysis (MLPA). Unrooted maximum-likelihood tree was based on concatenated sequences of five housekeeping genes (*gltA*, *gyrB*, *rpoB*, *tsf* and *zipA* genes), as described in Roger *et al*. [13]. Only type and references strains are indicated within clades on the tree. The horizontal lines show genetic distance, the scale bar indicating the number of substitutions per nucleotide position. The numbers at the nodes are support values estimated with 100 bootstrap replicates. Only bootstrap values >70 are indicated on the tree. When all members of a clade shared identical features, the common *rrn* operon number and/or PCR-TTGE pattern was indicated. * The *A. caviae* clade included the type strain *A. caviae* CECT 838^T^ displaying an external position to other members of the *A. caviae* clade in the tree.

When compared with multilocus phylotaxonomy, 23 of the 34 PCR-TTGE bands were specifically associated with all or some strains of a species while absent from all other taxa, indicating that some 16S rRNA gene copies can discriminate among *Aeromonas* species (Table 1 and Figure 1). The remaining 11 bands were shared by patterns of strains belonging to up to 8 taxa (e.g., bands 15 and 30) showing that strains from different species may harbor identical V3 region *rrs* copies. Diversity of patterns observed between strains belonging to a given species was mostly related to intragenomic V3 *rrs* copy heterogeneity. Among species represented by more than one strain, *A. caviae* and *A. media* showed the highest proportion of strains with heterogeneous TTGE patterns (76.5% and 66.7%, respectively), followed by *A. hydrophila*, *A. aquariorum* and *A. veronii* (48.6%, 37.5% and 29.6%, respectively) while none of the *A. salmonicida*, *A. sobria*, *A. allosaccharophila*, *A. piscicola*, *A. tecta*, and *A. jandaei* displayed *rrs* sequence heterogeneity (Table 1). The type strains of 7 other species displayed *rrs* V3 region sequence heterogeneity: *A. encheleia*, *A. enteropelogenes*/*trota*, *A. fluvialis*, *A. molluscorum*, *A. popoffii*, *A. sanarellii* and *A. taiwanensis* while no heterogeneity was found for the 7 remaining species *A. bestiarum*, *A. bivalvium*, *A. diversa*, *A. eucrenophila*, *A. rivuli*, *A. simiae*, and *A. schubertii*, as well as for the strains HG11 CECT 4253 and CCM 1271 (Table 1).

Regarding the 3 most represented species in our population, all *A. hydrophila* isolates harbored the band 1 that was associated or not with 10 different other bands (Table 1). Similarly, all *A. caviae* isolates shared the band 4 associated or not with 10 other bands. For *A. veronii*, band 11 was present in all the strains but one (strain AK241), associated or not with 6 other bands. *A. caviae* displayed the highest level of profile diversity, 17 patterns being observed among the 34 strains (0.5 pattern per strain) compared to *A. hydrophila* (11 patterns, 0.31 pattern per strain) and *A. veronii* (8 patterns, 0.11 pattern per strain) (Table 1). Heterogeneity frequency differed significantly between the three main species (*P* value = 0.00008), particularly when *A. caviae* was compared to *A. veronii* (*P* value = 0.000078) while difference was slightly significant between *A. hydrophila* and *A. veronii* (*P* value = 0.046). In spite of the low number of strains studied, it is noteworthy that none of the 6 isolates belonging to *A. media* shared an identical pattern (Table 1, Table S1) and that the 8 isolates of *A. aquariorum* showed a diversity of TTGE patterns similar to that of *A. caviae* (0.5 pattern per strain).

In addition to the high rate of *A. caviae* isolates with heterogeneous *rrs* copies, we observed among strains displaying V3 region *rrs* heterogeneity that TTGE profiles formed of 3 or 4 bands were significantly more frequent in *A. caviae* (16 out of 21 strains) than in other taxa (*P* value = 0.00048). These patterns were particularly found in *A. caviae* isolated from human clinical samples (12 of the 16 strains), mainly from gut-related specimens (10 out of 12 strains). However, strains recovered from other origins were underrepresented among the *A. caviae* isolates of our collection (10 strains from non-human and environmental samples, and 6 strains from human specimens not related to the gut) (Table S1). Identical complex PCR-TTGE patterns, e.g., 1+4+18 for *A. caviae* strains BVH63 and BVH84 and 3+4+11 for *A. caviae* strains BVH19 and BVH4 were observed in strains isolated from geographically distant French regions. Another outstanding example is represented by the pattern 3+4+18 observed for the two environmental *A. caviae* strains CCUG 48892 isolated from water in Sweden in 2004 and AK234 recovered from wastewater treatment lagoon in France in 2006. Beside *A. caviae* members, the strains displaying 3- or 4-band TTGE patterns were *A. molluscorum* and *A. enteropelogenes* type strains, one *A. hydrophila* strain from human wound and two *A. media* strains from snail and oyster (Table S1).

### High *rrn* Copy Number with Low Structural Variability in Aeromonads

PFGE patterns were obtained for 189 strains giving for the first time a whole perspective of *rrn* copy number and distribution across the chromosome for about 20 not yet investigated *Aeromonas* species, as well as intrapecific variability data. PFGE patterns obtained for all the type and reference strains of this study are shown in Figure S2. The population displayed 8 to 11 I-*Ceu*I-restricted DNA fragments corresponding to 8 to 11 *rrn* operons. However, the variability in the whole genus was low, more than 92% of the strains displaying 10 *rrn* operons (n = 175). Species and/or strains harboring 8 (n = 3), 9 (n = 8) or 11 (n = 3) *rrn* operons were rare and no particular feature could be identified for these bacteria being either recovered from human (n = 5), non-human (n = 6) or environmental (n = 3) samples (Table S1). Intraspecific variability in *rrn* operon number was observed for *A. salmonicida*, *A. aquariorum, A. caviae* and *A. veronii* strains (Figure 1, Table S1) but the most notable variability was observed for *A. salmonicida* and *A. aquariorum* with 50% and 25% of the strains harboring 9 *rrn* operons, respectively. Finally, no correlation could be drawn here between *rrn* operon number and *rrs* V3 region heterogeneity due to the low number of strains harboring 8, 9 or 11 operons.

**Figure 2:**
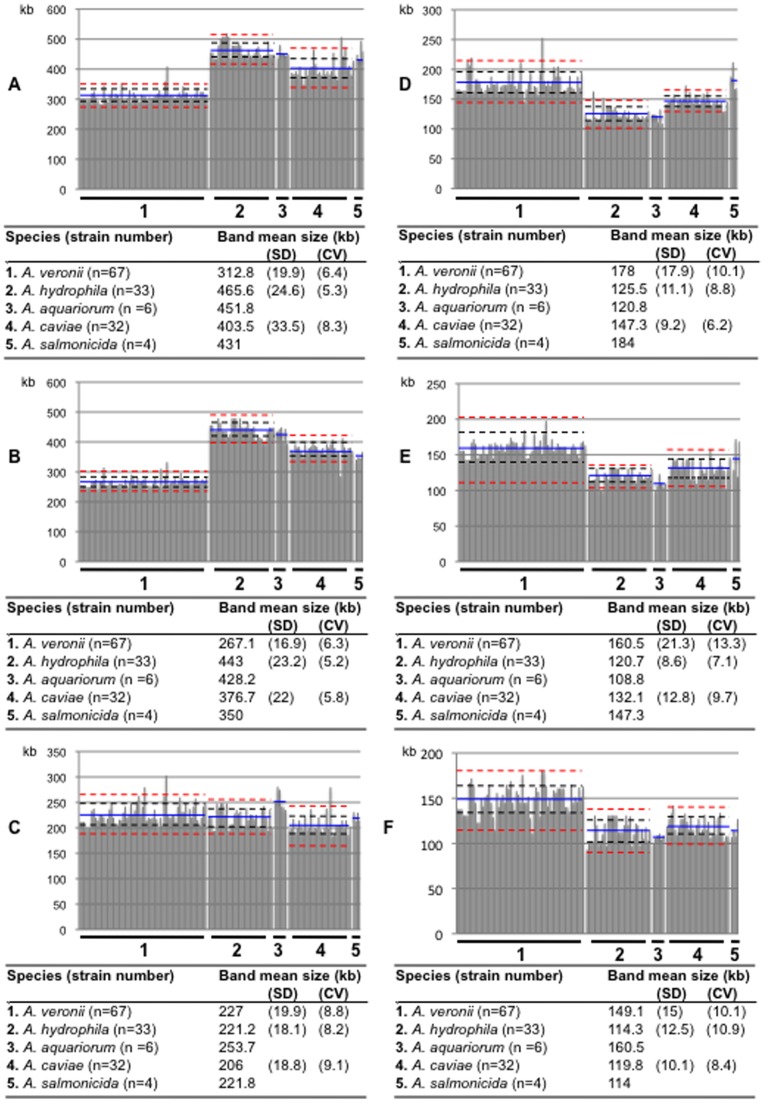
Schematic representation of I-*Ceu*I-restricted DNA fragment sizes for *Aeromonas* spp. strains with 10 *rrn* operons. Data were given for the 142 *A. veronii*, *A. hydrophila*, *A. aquariorum*, *A. caviae* and *A. salmonicida* strains with 10 *rrn* operons with the aim to illustrate interspecific variability between the 5 taxa and the intraspecific variability for the 3 main species. I-*Ceu*I-restricted DNA fragments were numbered in size descending order; the size of the largest fragment 1 was not measured (as discussed in the text). A to F, size distribution for fragments 2 to 7, respectively. Fragment mean sizes (in kilobases) were indicated in the corresponding tables according to the species and by a blue line in the corresponding scheme A to F. Standard deviation values (SD) and coefficient of variation (CV) were indicated in kb and %, respectively, in the corresponding tables according to the species except for species with less than 10 representatives; +/−1 SD were represented by black dotted lines and +/−2 SD by red dotted lines in the corresponding scheme A to F. Schematic representations for the 3 smallest fragments with mean size lower than 97 kb (mean size and SD of 93.7±23 kb, 66.7±9.1 kb and 40.5±7.5 kb, respectively) were not presented here because they were not informative.

Beside variability in copy number, variability in size of I-*Ceu*I fragments, i.e., variability in location of *rrn* on the chromosome, was also observed in the population. Variability in the size of the largest I-*Ceu*I fragment could not be precisely measured using the migration conditions applied herein. Considering the 3 complete genome sequences, the largest fragment size variation was 2950±70 kb (2.4%) (3019 kb for the *A. hydrophila*, 2885 kb for the *A. salmonicida* and 3001 kb for the *A. veronii* strains), a value in agreement with sizes previously evaluated to 2900 kb for *A. hydrophila* strain JMP636 [25] using PFGE-based experiment. Based on this low variability in size, we assumed that excluding this fragment from pattern comparison would not greatly affect both intra- and interspecific diversity analysis. Despite this limitation, we showed that most *Aeromonas* species had a quite specific I-*Ceu*I fingerprint with exception of *A. aquariorum*, which displayed a roughly similar pattern to the one observed for *A. hydrophila* (Figure 2, Figure S2, Table S1). Another notable exception was *A. media* that displayed highly diverse *rrn* chromosomal repartition (Figure S2, Table S1). We also evaluated the variability of *rrn* chromosomal distribution at the species level for the main species represented by a large number of strains, *A. hydrophila*, *A. caviae* and *A. veronii* (Figure 2). For the 6 genomic fragments analyzed, the coefficient of variation of the mean size varied from 5.2% to 13.3%. The fragment with the most variable size varied according to the species. *A. veronii* was the most variable species for the size of fragments B, D and E, *A. caviae* for fragments A and C, and *A. hydrophila* for fragment F. Regardless of the species, A and B fragments appeared the most stable in size. Despite this intraspecific variability, the size of most genomic fragments enabled to distinguish a species from others (Figure 2).

### RiMOD Fits the Taxonomy of Aeromonads

RiMOD has been confronted to the current taxonomy of the genus *Aeromonas* including recent taxonomic changes. Firstly, several recently characterized species such as *A. taiwanensis*, *A. sanarellii* and *A. fluvialis* [2], [3] displayed clearly distinct features in both PFGE and TTGE-based analyses (Table S1, Figure 1, Figure S2). The proposal of *A. diversa* including *Aeromonas* sp. HG13, so-called *Aeromonas* group 501, as a distinct species from *A. schubertii* [26] was supported in PCR-TTGE by different *rrs* V3 regions. These 2 species displayed 8 *rrn* operons, as observed for the MLPA-related species *A. simiae* (Figure 1), but their PFGE fingerprints were clearly individualized (Table S1, Figure S2). Moreover, the suggestion that *A. hydrophila* subsp. *anaerogenes* and *A. caviae* are conspecific [27] was supported by the RiMOD results, *A. hydrophila* subsp. *anaerogenes* strain CECT 4221 displaying a *rrn* operon PFGE fingerprint related to those observed in members of the *A. caviae* clade and the 3+4 PCR-TTGE pattern never observed in the *A. hydrophila* clade members (Figure 1, Figure S2).

Several taxonomic reappraisals were supported by one method of the RiMOD approach. In particular, we distinguished *A. hydrophila* from *A. aquariorum*, a species proposed in 2008 on the basis of *gyrB* and *rpoD* gene phylogeny to accomodate members of the subspecies *A. hydrophila* subsp. *dhakensis* [28]. Although PFGE fingerprinting did not distinguish *A. aquariorum* from *A. hydrophila*, PCR-TTGE clearly showed that *rrs* V3 region was discriminative between the 2 species, band 3 being found in all *A. aquariorum* isolates while absent from all the *A. hydrophila* strains (Table 1, Figures 1 and 2, Table S1, Figure S2).

Additional arguments were also given here on controversial issues recently reviewed by Janda & Abbott [29]. *A. ichthiosmia* and *A. culicicola* considered as later synonyms of *A. veronii* [30] shared similar PFGE patterns and displayed PCR-TTGE patterns mainly (pattern 11) or exclusively (pattern 4+11) observed in the *A. veronii* clade. (Table S1, Figure S2). Previous suggestions to unify the two pairs of taxa *A. enteropelogenes* and *A. trota* on one hand, and *A. encheleia* and HG11 on the other hand could hardly be addressed and discussed here because one strain only for each taxon was included in the study. We noted that *A. enteropelogenes* and *A. trota* displayed closely related *rrn* operon PFGE fingerprints and specific TTGE patterns, i.e., 3+4+36 and 4+15, respectively, not shared by any other strain of the collection despite all bands forming these patterns with the exception of the band 36 were present in other species. Similarly, *A. encheleia* and strain representative of HG11 respectively displayed TTGE patterns 1+30 and 15+30 formed by bands found in other taxa but showed distinct *rrn* operon PFGE patterns (Table 1, Figure S2). For *A. allosaccharophila*, our results supported the unification of the species with *A. veronii* as suggested by DNA-DNA hybridization experiment and contrarily to MLPA results because strains shared similar *rrn* operon PFGE patterns and harbored main TTGE bands differing by a unique variable position (band 13 in the 3 *A. allosaccharophila* strains and band 11 found in 70 out of the 71 *A. veronii* isolates of the study) (Table 1, Figure 1). Of note, the only *A. veronii* isolate lacking band 11 had a PCR-TTGE pattern 13. Although the number of *A. allosaccharophila* strains studied in this work was too low to be conclusive, these observations together with previous results leading to controversy on the taxonomic status of this species [29] may suggest that *A. allosaccharophila* is at the starting time of speciation.

In addition, previous MLPA results suggested that the species *A. media* could be polyphyletic containing yet-undescribed taxa [13] and that strain CCM 1271 probably represent an unknown *Aeromonas* taxon [11], [13]. Here, we confirmed the heterogeneity of genetic and genomic features of the 6 studied *A. media* strains (Figure 1, Table S1, Figure S2) and showed that strain CCM 1271, referenced in the Czech Collection of Microorganisms as *A. bestiarum*, shared the TTGE pattern 30 with *A. bestiarum*, *A. salmonicida* and *A. piscicola* (Table 1) but presented a PFGE fingerprint distinct from those observed for these 3 species by the number and/or the chromosomal repartition of *rrn* operons (data not shown).

Results obtained for *A. sharmana*, a species further shown not to belong to the genus *Aeromonas* [31], [32] confirmed its distant relationship to members of the studied population by showing a specific and unique PCR-TTGE band (data not shown) and a *rrn* skeleton not clearly resolved in the conditions appropriate to the study of *Aeromonas* spp. but confidently formed by 6 or 7 operons (Figure S2).

## Discussion

### Striking Diversity in Ribosomal Genes of Aeromonads

We described an outstanding diversity in ribosomal genes in a representative population of aeromonads. This result is paradoxal considering that the majority of species in the genus shared more than 99% of their nucleotide positions when 16S rRNA gene is directly sequenced from whole genomic DNA. The diversity described herein concerns both the number and the polymorphism of *rrs* copies and is therefore underestimated when the whole 16S rRNA gene sequence that reflects the mix of sequences from a multigenic family is studied. Indeed, we confirmed on a large panel of strains the high *rrn* copy number in *Aeromonas* spp. previously published, i.e., 9 operons in most *A. salmonicida* [16], [18] and 10 operons in *A. hydrophila* subsp. *hydrophila* strain ATCC 7966^T^ [17], *A. culicicola* strain MTCC 3249^T^ [33] and *A. veronii* strain B565 [19].

The *rrn* operons in aeromonads are to be considered as a multi-operon family given the number of operon copies and their diversity. As shown in a previous study, we confirmed that PCR-TTGE is a powerful approach to study the rate of bacterial isolates with *rrs* heterogeneity in large natural populations [24]. In this previous study conducted on *Veillonella* spp. that displayed 4 *rrn* copies, all the bands observed in TTGE were successfully sequenced after a single run of PCR-TTGE [24]. For aeromonads, interpretable sequences were obtained for 44% of the bands only after a single run of PCR-TTGE and 15% of the bands remained unsequenced after a double run. We assumed that this was related to the high number of *rrn* operons in aeromonads leading to band cross-contamination during the electrophoresis step and thereby constituted a limitation of the method when applied to bacteria harboring a high number of *rrn* operons. Sequences obtained for 29 TTGE bands showed up to 13 variable positions between *rrs* V3 region copies in a genome, a number exceeding the 1 to 10 microheterogeneities (0.06 to 0.66% of divergence) observed through chromatograms of the nearly complete 16S rRNA gene sequences by Alperi *et al*. [10] and the number of divergent nucleotides (2 to 5) observed between *rrs* copies for the 3 strains with complete genome sequences.

V3 *rrs* region analysis revealed an unexpectedly high rate of *Aeromonas* strains displaying *rrs* intragenomic heterogeneity compared with previously published studies based on other methodological approaches [9], [10]. Indeed, we showed that 41.5% of the strains included in our study displayed *rrs* V3 region heterogeneity while microheterogeneities were suspected from atypical RFLP patterns in only 8.1% of the 999 *Aeromonas* strains examined by Alperi *et al*. [10], and in 21% of the 82 strains studied by Morandi *et al*. [9]. We suspected this rate to be even higher because the PCR-TTGE approach is limited by the size of the amplified fragment that can migrate in TTGE, i.e., approximately 200 bp. Although the V3 region is one of the most variable regions among the 16S rRNA gene [34], intragenomic heterogeneity may still be underestimated when the V3 region alone is studied as shown by the *in silico* analysis of the three totally sequenced genomes. Indeed, *rrs* microheterogeneity was observed in the 3 strains, *A. hydrophila* subsp. *hydrophila* strain ATCC7966^T^ harbored 3 different *rrs* copies among 10 resulting from up to 2 divergent nucleotides; *A. veronii* strain B565 harbored 5 different *rrs* copies among 10 (3 divergent nucleotides), and *A. salmonicida* subsp. *salmonicida* strain A449 showed the highest level of intragenomic heterogeneity with 5 distinct *rrs* copies among 9 differing by up to 5 nucleotides. Using V3 region *rrs* PCR-TTGE, intragenomic heterogeneity would have been observed in the *A. salmonicida* strain only, one of the 5 polymorphic nucleotides being located in the V3 region while overlooked in the other two strains because none of the divergent nucleotides were located in the targeted variable region. This was confirmed here by the one band PCR-TTGE pattern observed for the type strain of *A. hydrophila* subsp. *hydrophila* purchased from another strain collection. Of note, *A. salmonicida* complete genome sequence showed the existence of *rrs* heterogeneity within the V3 region for strain A449 not detected in strains included in our study.

Diverse RFLP patterns have been observed for *A. veronii*, *A. media* and *A. encheleia* in a study investigating 62 reference strains belonging to 13 species while other species included in the study were thought to harbor conserved 16S rRNA genes [7]. Further studies suspected or demonstrated that 16S rRNA gene intragenomic heterogeneity is encountered in a wider range of species within the genus (for a review, see [10]). We demonstrated that intragenomic *rrs* heterogeneities occurred in half of the taxa included in our study and firstly described the occurrence of such microheterogeneities in the recently described taxa *A. fluvialis*, *A. sanarelli*, and *A. taiwanensis*. We also confirmed microheterogeneities previously suspected in some taxa such as *A. trota* [10], [35]. The rate of isolates displaying diversity of the *rrs* V3 region copies varied according to the taxon considered. Among the 3 main clades, *A. caviae* displayed the highest number of isolates with V3 *rrs* heterogeneity in agreement with a previous RFLP-based study [10]. However, we found that *A. media* was the species displaying the highest intraspecific *rrs* V3 region diversity, each of the 6 strains displaying its own pattern, before *A. aquariorum*, *A. caviae*, *A. hydrophila* and then *A. veronii*, thereby differing from the distribution observed by Alperi *et al*. after RFLP patterns analysis [10].

### RiMOD and Aeromonad Evolution

*rrn* distribution across the chromosome, mainly stable within and different between species, reflected evolutionary relationships between aeromonads. Among the 3 main clades, patterns observed for *A. hydrophila* and *A. caviae* were more similar while those observed for *A. veronii* were more distinct and this reflected the relationships observed in the MLPA-based tree. In contrast, closely related species in the MLPA-based tree that may represent more recently emerging species, such as *A. allosaccharophila* compared to *A. veronii* and *A. aquariorum* compared to A. *hydrophila*, were indistinguishable using this approach. This suggested that comparison of I-*Ceu*I fingerprints may reflect phylogenetic relationships in the genus *Aeromonas* and allow recognition and/or delineation of species complexes, from which novel lineages or novel species may emerge.

It is generally admitted that intragenomic 16S rRNA gene heterogeneity is due to mutations after gene duplication or to horizontal gene transfer (HGT) [36], [37], followed by a lack of intragenomic concerted evolution [38]. In the genus *Streptomyces*, it has been suggested that copies differing by one or two nucleotides originate from mutations while copies differing by more than 5 nucleotide positions and found in another species more likely originate from HGT [39]. Besides, the occurrence of hybrid events in 16S rRNA gene sequences was previously demonstrated in the form of gene crossovers [9], [40].

All together, the observation of species-specific bands, the recovery of identical V3 region in different species and intragenomic heterogeneity of *rrs* V3 copies differing by one to 13 nucleotides argue for the existence of the two modes of *rrs* evolution in the genus *Aeromonas*. As an example, bands 3 and 18 found in some *A. caviae* strains, each differing by one nucleotide from the band 4 found in all *A. caviae* strains, were probably generated by mutation while band 1 differing by 12 nucleotides from band 4 was probably transferred to *A. caviae* by HGT. We showed that *rrs* V3 region HGT defined by more than 5 divergent nucleotidic positions, was detected in 23 PCR-TTGE patterns observed in 7 species (Table S1). Our results also supported a non-random occurrence of some particular heterogeneous patterns because unrelated isolates shared identical patterns made of a combination of bands generated by mutations or by mutations and HGT, e.g., patterns 3+4+18 and 1+4+18 in *A. caviae*.

It is noteworthy that the concerted evolution as a mode of homogenization of copies in multigenic families is not efficient for most strains in aeromonads. Homogenization of copies is a spontaneous mechanism that occurs by homologous recombination among closely related sequences. The rate of isolates harboring divergent *rrs* copies in aeromonads suggested a selective pressure that maintains differences in their sequences. This is also the case for the maintenance of a high number of *rrn* operons in spite of the associated metabolic cost [41].

### RiMOD and Lifestyles in Aeromonads

Harboring about 10 *rrn* operons is highly atypical compared with bacteria in general and particularly with other well-known waterborne human opportunistic pathogens such as *Pseudomonas aeruginosa* for which genome sequencing revealed 4 *rrn* operons only. According to the ribosomal RNA operon copy number database (rrndb, consulted on February the 16th, 2012) [42], 8 to 11 operons have been reported in only 46 out of 782 species (6%). Focusing on bacteria harboring 10 *rrn* operons other than *Aeromonas* revealed 13 species distributed among 7 genera and clustering in few phylogenetic lineages, i.e., 6 families (*Clostridiaceae*, *Peptococcaceae*, *Bacillaceae*, *Aeromonadaceae*, *Psychromonadaceae* and *Shewanellaceae*) belonging to 2 phyla (*Firmicutes* and *Proteobacteria*). Within the class *Gammaproteobacteria*, *Aeromonas* spp. shared common characteristics with members of the genus *Shewanella* (8 to 11 *rrn* operons recorded among the 16 entries available in the rrndb for the genus), a genus found in highly diverse aquatic environments and considered as a human emerging opportunistic pathogen [43]–[45]. It has been proposed that sequence modifications among 16S rRNA gene copies provide a fine-tuning of the ribosome function to optimize bacterial niche fitness [46]. Similarly, it could be hypothesized that a high number of 16S rRNA gene copies and a high rate of isolates with intragenomic heterogeneity could be features involved in maintaining functional diversity of *Aeromonas* spp. in environment, animal and human microbiotae.

Here, *A. caviae* displayed outstanding characteristics, i.e., the highest number of isolates with V3 *rrs* heterogeneity and the highest diversity in the heterogeneity types. These features significantly different from those observed in other species may be related to a particular lifestyle and an ongoing process of adaptation of this species to a particular niche that could be represented, as previously hypothesized, by the gut [13]. It was also noteworthy that among the 71 *A. veronii* studied strains, the 22 *A. veronii* isolates displaying *rrs* heterogeneity were all recovered from human samples except for two strains recovered from animal samples while all strains isolated from environmental specimens (n = 20) had identical *rrs* V3 region copies. More generally, we showed that isolates recovered from human samples more frequently displayed V3 *rrs* heterogeneity than isolates recovered from samples from non-human origin according to previous results of Alperi *et al.* [10].

RiMOD data confirmed and completed the vision of aeromonads as a bacterial population structured in species complexes, an organization associated with bacteria displaying sympatric lifestyles, i.e., a high level of horizontal gene transfers, a large pan-genome, a large genome, and a significant number of ribosomal operons, and a behavior of generalist opportunistic pathogen [21].

### Taxonomic Considerations

Taxonomy of the genus *Aeromonas* has greatly evolved over the past decades following advances in methods applied to infer relationships between members of the genus. The bulk 16S rDNA sequencing was shown to be insufficiently discriminatory between tightly related species [1]–[4]. The use of 16S rRNA gene PCR-RFLP has then been proposed for species identification [5], [6]. However, considering the endonucleases used in 16S rRNA gene PCR-RFLP, the intragenomic divergent positions identified in this study within the *rrs* V3 region modified the number of *Alu*I restriction sites in 16% of the strains (32 strains belonging to 7 taxa and displaying 15 different TTGE patterns), as well as the number of *Nar*I restriction sites in the type strain of *A. encheleia* (data not shown), thereby leading to atypical restriction patterns and possible misidentification of the strains as also previously underlined [7]–[10]. Thus, the high intragenomic heterogeneity of the different *rrs* sequences and the intraspecific *rrs* variability, sometimes exceeding the interspecific variability, blurred the 16S rRNA-based studies that were progressively replaced or implemented by single housekeeping gene analysis and further by multilocus sequence analysis [2]–[4], [11]–[13]. However, some conflicting results may arise from the use of different approaches. For example, *A. allosaccharophila* existence is still controversial despite DNA-DNA hybridization values to *A. veronii* strain CECT 4247^T^ ( = ATCC 35624^T^) have been reevaluated to 78–82% by Huys *et al*. [30]. Based on single housekeeping gene (*gyrB*, *cpn60*, *tsf* or *zipA*)-based studies, the taxon occupied a closely related but robust external position to the *A. veronii* clade supporting the separation of the two taxa [13], [47], [48]. Similar results have been observed with MLPA studies [11], [13]. Recently, a hypothesis that could support at least in part some controversial taxonomic data was proposed based on the evolution mode of *Aeromonas* spp. Some taxa subjected to controversy may correspond to lineages emerging from species complexes and for which signs of starting speciation are detected or not according to the characteristic investigated and to the method used [13], [21], [49]. RiMOD study supported this hypothesis for *A. allosaccharophila*.

Here, we showed that *rrs* heterogeneity patterns might inform on taxonomic relationships between species because some PCR-TTGE bands were specific of some species despite HGT. On the other hand, large-scale chromosome structure has previously been described as a sensitive indicator of phylogenetic relationships between bacteria [50]–[53] but it received little attention in the polyphasic strategy used for taxonomic studies in the genus *Aeromonas*. In this study, *rrn* operon number at 8 as well as *rrn* operon PFGE fingerprints were valuable for taxonomic purpose, allowing taxonomic resolution of species complexes. To our knowledge, only the study by Umelo & Trust on *A. salmonicida* previously showed that I-*Ceu*I digestion fingerprints could help identify related strains and thus help to better classify and subdivide the atypical strains [16]. Combining both approaches for studying RiMOD supported current taxonomy including recent taxonomic changes in the genus *Aeromonas* and was especially useful here to identify heterogeneity within MLPA clades such as *A. salmonicida* and *A. media* clades. The latter species warrant further investigations to search whether they are robust groups including atypical strains or groups submitted to a tempo of evolution associated to a high rate of speciation or a strong selective pressure generating diversity within the group, or polyphyletic taxa. We therefore assumed that RiMOD approach proposed herein could be useful to complement the polyphasic strategy used for isolate characterization within the genus *Aeromonas*.

The 16S rRNA gene is often criticized because this marker provides a limited point of view on the bacterial evolution. However, 16S rRNA-based hierarchical system is considered to be the backbone of prokaryote taxonomy that was globally confirmed by phylogenomics. The main pitfalls of 16S rRNA-based phylogeny is the low amount of genetic information explored regarding genome size and the multigenic organisation that lead to blurred information when bulk DNA is sequenced. However, multigene family is an exception in bacterial genome and therefore the maintaining of several heterogeneous 16S rRNA loci as observed in aeromonads is probably not trivial. In this study, we showed that 16S rRNA multigenic organisation should no longer be considered as a pitfall that confuse relationships among aeromonads but rather be exploited in all the dimensions of its diversity, which probably reflects major mechanisms involved in the generalist pathogen lifestyle [21], as previously observed for other *Aeromonas* genetic and genomic traits [54].

## Supporting Information

Figure S1 **TTGE profiles of amplified 16S rRNA gene V3 region for** ***Aeromonas*** **spp. strains. A)** Lanes 1–12, *A. veronii* isolates ADV103, BVH61, ADV109, ADV119, ADV125, ADV127, ADV129, ADV130, ADV131, ADV133, ADV135, and ADV137b; lane 13, *A. bivalvium* CECT 7113^T^. Profiles are indicated at the bottom of the figure. Arrows indicated the position of the four TTGE bands constituting the *A. molluscorum* type strain pattern. **B)** 13 *Aeromonas* spp. type and reference strains. Names of strains are indicated at the top of the figure. Each TTGE band number is noted on the band. L, ladder with 1+4+15+30 profile.
(TIF)

Figure S2 **PFGE migration of genomic DNA digested by I-** ***Ceu*** **I for** ***Aeromonas*** **spp. strains. A)** 37 type and reference strains of *Aeromonas* spp. and *A. sharmana* DSM 17445^T^. Names of strains and names of main clades are indicated at the top and bottom of the figure, respectively. Lambda ladder was used as molecular size marker (lanes M). Sizes are indicated in kilobases (kb). **B)** Lane 1, *A. hydrophila* subsp. *dhakensis* CIP 107500 (now *A. aquariorum*); lane 2, *A. hydrophila* subsp. *hydrophila* CECT 839^T^; lane 3, *A. hydrophila* subsp. *ranae* CIP 107985; lane 4, *A. aquariorum* CECT 7289^T^; lane 5, *A. caviae* strain ADV118; lane 6, *A. caviae* strain ADV121; lane 7, *A. caviae* CECT 838^T^; lane 8, *A. media* CECT 4232^T^; lane 9, *A. allosaccharophila* CECT 4199^T^; lane 10, *A. culicicola* CIP 107763^T^ (now *A. veronii*); lane 11, *A. ichtiosma* CECT 4486^T^ (now *A. veronii*); lane 12, *A. veronii* biovar *veronii* CECT 4247^T^; lane 13, *A. veronii* biovar *sobria* LMG 13067; lane 14, *A. media* strain BVH40; lane 15, *A. media* strain AK202; lane 16, *A. media* strain AK211. Lambda ladder was used as molecular size marker (lanes M). Sizes are indicated in kilobases (kb).
(TIF)

Table S1 **PCR-TTGE pattern,** ***rrs*** **V3 region heterogeneity and** ***rrn*** **operon number for the 195** ***Aeromonas*** **strains of the study according to multilocus phylogenetic clade determined in Roger** ***et al*** **.** [**13**] **.**
(DOCX)
