# Supplementary figures and images for: Ribosomal Multi-Operon Diversity: An Original Perspective on the Genus Aeromonas
Source: PLoS One. 2012 Sep 27;7(9):e46268. doi: 10.1371/journal.pone.0046268 (PMC3459834; doi:10.1371/journal.pone.0046268)

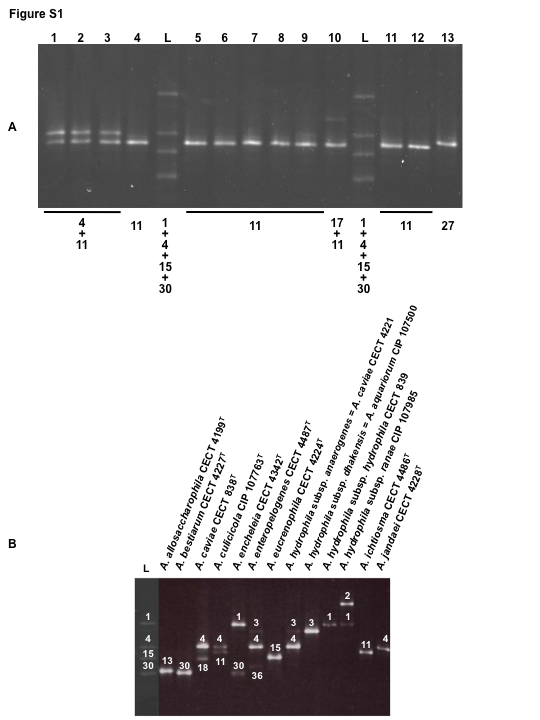

Supplement: Figure S1 — TTGE profiles of amplified 16S rRNA gene V3 region for Aeromonas spp. strains. A) Lanes 1–12, A. veronii isolates ADV103, BVH61, ADV109, ADV119, ADV125, ADV127, ADV129, ADV130, ADV131, ADV133, ADV135, and ADV137b; lane 13, A. bivalvium CECT 7113T. Profiles are indicated at the bottom of the figure. Arrows indicated the position of the four TTGE bands constituting the A. molluscorum type strain pattern. B) 13 Aeromonas spp. type and reference strains. Names of strains are indicated at the top of the figure. Each TTGE band number is noted on the band. L, ladder with 1+4+15+30 profile. (TIF) [file pone.0046268.s001.tif]
