# Supplementary material for: Ribosomal Multi-Operon Diversity: An Original Perspective on the Genus Aeromonas
Source: PLoS One. 2012 Sep 27;7(9):e46268. doi: 10.1371/journal.pone.0046268 (PMC3459834; doi:10.1371/journal.pone.0046268)
Supplement: Table S1 — PCR-TTGE pattern, rrs V3 region heterogeneity and rrn operon number for the 195 Aeromonas strains of the study according to multilocus phylogenetic clade determined in Roger et al . [13] . (DOCX) [file pone.0046268.s003.docx]

**Table S1.** PCR-TTGE pattern, *rrs* V3 region heterogeneity and *rrn* operon number for the 195 *Aeromonas* strains of the study according to multilocus phylogenetic clade determined in Roger *et al*. [13].

| **MLPA clade or isolated taxon ^a^** | **Strain** | **PCR-TTGE**  **pattern ^b^** | ***rrs* V3 region**  **heterogeneity level**  **(nt number / similarity %) ^c^** | ***rrn* operon copy number** | **I-*Ceu*I-digested fragment sizes after PFGE ^d^** | | **Origin ^e^** | | **Region, country and year of isolation** |
| --- | --- | --- | --- | --- | --- | --- | --- | --- | --- |
| ***A. veronii***  **(n=71)** | BVH22 | 11+15 | 8 nt / 94.7% | 10 | X – 300 – 255 – 212 – 166 – 156 – 138 – 104 – 54 - 39 | | Human, Wound | | Alès, Fr, 2006 |
|  | BVH23 | 11+15 | 8 nt / 94.7% | 10 | X – 300 – 255 – 212 – 166 – 156 – 138 – 104 – 54 - 39 | | Human, Wound | | Saint-Brieux, Fr,2006 |
|  | BVH25b | 11+15 | 8 nt / 94.7% | 10 | X – 300 – 255 – 212 – 166 – 156 – 138 – 104 – 54 – 39 | | Human, Respiratory tract | | Saint-Brieux, Fr,2006 |
|  | BVH26a | 11+15 | 8 nt / 94.7% | 10 | X – 298 – 249 – 200 – 164 – 143 – 131 – 97 – 54 – 39 | | Human, Wound | | Saint-Brieux, Fr,2006 |
|  | BVH27a | 11+15 | 8 nt / 94.7% | 10 | X – 298 – 249 – 200 – 164 – 143 – 131 – 97 – 54 – 39 | | Human, Wound | | Reunion Island, Fr,2006 |
|  | BVH28a | 11+15 | 8 nt / 94.7% | 10 | X – 298 – 249 – 200 – 164 – 143 – 131 – 97 – 54 – 39 | | Human, Wound | | Reunion Island, Fr,2006 |
|  | BVH61 | 4+11 | 5 nt / 96.7% | 10 | X – 320 – 266 – 234 – 216 – 167 – 167 – 115 – 67 – 38 | | Human, Stool | | Antibes, Fr,2006 |
|  | BVH71 | 4+11 | 5 nt / 96.7% | 10 | X – 327 – 270 – 238 – 206 – 172 – 172 – 120 – 75 – 47 | | Human, Stool | | Martinique Island, Fr, ND |
|  | BVH47 | 4+11 | 5 nt / 96.7% | 10 | X – 306 – 275 – 220 – 220 – 165 – 165 – 114 – 60 – 29 | | Human, Blood | | Roubaix, Fr,2006 |
|  | ADV102 | 4+11 | 5 nt / 96.7% | 10 | X – 295 – 242 – 214 – 163 – 145 – 145 – 100 – 57 – 41 | | Human, Stool | | Montpellier, Fr, 2008 |
|  | BVH18 | 11 | - | ND | ND | | Human, Wound | | Villeneuve sur Lot, Fr, 2006 |
|  | AK249 | 11 | - | ND | ND | | Environment,  Water lake | | Annecy, Fr, 1998 |
|  | ADV129 | 11 | - | 10 | X – 283 – 269 – 226 – 183 – 151 – 123 – 95 – 58 – 34 | | Human, Stool | | Montpellier, Fr, 2009 |
|  | ADV133 | 11+17 | ND | 10 | X – 283 – 283 – 236 – 188 – 162 – 134 – 95 – 68 – 44 | | Human, Wound | | Montpellier, Fr, 2010 |
|  | BVH90 | 11 | - | 10 | X – 346 – 315 – 222 – 180 – 163 – 150 – 128 – 76 – 57 | | Human, Stool | | Dunkerque, Fr, 2006 |
|  | AK236 | 11 | - | 10 | X – 303 – 247 – 207 – 168 – 150 – 141 – 111 – 60 – 40 | | Environment, Water lake | | Annecy, Fr, 1998 |
|  | BVH37 | 11 | - | 10 | X – 291 – 255 – 218 – 160 – 151 – 151 – 103 – 54 – 34 | | Human, Blood | | La Roche sur Yon, Fr, 2006 |
|  | BVH46 | 11 | - | 10 | X – 315 – 266 – 236 – 165 – 165 – 154 – 103 – 67 – 24 | | Human, Blood | | Roubaix, Fr, 2006 |
|  | BVH56 | 11 | - | 10 | X – 331 – 272 – 248 – 174 – 165 – 165 – 140 – 73 – 49 | | Human, Blood | | Versailles, Fr, 2006 |
|  | ADV101 | 11 | - | 10 | X – 303 – 254 – 220 – 169 – 145 – 145 – 100 – 57 – 41 | | Human, Stool | | Montpellier, Fr, 2008 |
|  | *A. veronii* bv. *veronii* CECT 4257^T^ | 11 | - | 10 | X – 318 – 256 – 205 – 188 – 167 – 129 – 97 – 63 – 39 | | Human, Respiratory tract | | Michigan, USA, NA |
|  | *A. veronii* CCM 4360 | 11 | - | 10 | X – 314 – 256 – 207 – 175 – 164 – 115 – 103 – 67 – 67 | | Human, Stool | | Connecticut, USA, 1984 |
|  | BVH6 | 11 | - | 10 | X – 300 – 260 – 216– 175 – 156 – 156 – 94 – 49 – 34 | | Human, Wound | | Cahors, Fr, 2006 |
|  | BVH13 | 11 | - | 10 | X – 300 – 260 – 216– 162 – 147 – 147 – 94 – 49 – 34 | | Human, Blood | | Le Mans, Fr, 2006 |
|  | BVH26b | 11 | - | ND | ND | | Human, Wound | | Saint-Brieux, Fr,2006 |
|  | BVH31 | 11 | - | 10 | X – 355 – 260 – 215– 177 – 165 – 140 – 140 – 60 – 34 | | Human, Bile | | La Rochelle, Fr, 2006 |
|  | BVH32 | 11 | - | 10 | X – 291 – 255 – 230– 160 – 160 – 148 – 123 – 54 – 34 | | Human, Stool | | La Rochelle, Fr, 2006 |
|  | BVH44 | 11 | - | 10 | X – 291 – 276 – 242– 171 – 154 – 154 – 114 – 60 – 29 | | Human, Wound | | Périgueux, Fr, 2006 |
|  | BVH49 | 11 | - | 10 | X – 306 – 248 – 220– 174 – 165 – 145 – 114 – 49 – 29 | | Human, Stool | | Chalon sur Saone, Fr, 2006 |
|  | BVH50 | 3+11 | 6 nt / 96% | 10 | X – 331 – 266 – 226– 174 – 165 – 165 – 108 – 60 – 29 | | Human, Respiratory tract | | Chalon sur Saone, Fr, 2006 |
|  | **BVH53** | 11 | - | 10 | X – 291 – 291 – 248– 174 – 174 – 160 – 146 – 67 – 29 | | Human, Blood | | Saint Denis, Fr, 2006 |
|  | BVH54 | 11+16 | 1 nt / 99.3% | 10 | X – 323 – 278 – 226– 194 – 169 – 169 – 114 – 79 – 60 | | Human, Respiratory tract | | Saint Denis, Fr, 2006 |
|  | BVH59 | 11+17 | ND | 10 | X – 314 – 256 – 234– 176 – 167 – 145 – 128 – 75 – 47 | | Human, Blood | | Le Havre, Fr, 2006 |
|  | BVH60 | 11+33 | ND | 10 | X – 388 – 320 – 252– 202 – 167 – 137 – 137 – 63 – 42 | | Human, Stool | | Antibes, Fr, 2006 |
|  | BVH73 | 11 | - | 10 | X – 302 – 280 – 252– 194 – 167 – 167 – 154 – 75 – 47 | | Human, Blood | | Martinique Island, Fr, NA |
|  | BVH77 | 11+17 | ND | 10 | X – 291 – 263 – 263– 172 – 158 – 158 – 120 – 97 – 49 | | Human, Stool | | Aix en Provence, Fr, 2006 |
|  | BVH79 | 11 | - | 10 | X – 315 – 268 – 223– 167 – 160 – 154 – 116 – 97 – 49 | | Human, Wound | | Aix en Provence, Fr, 2006 |
|  | BVH80 | 11+15 | 8 nt / 94.7 % | 10 | X – 312 – 274 – 242– 180 – 167 – 145 – 123 – 73 – 53 | | Human, Stool | | Aix en Provence, Fr, 2006 |
|  | BVH95 | 11+15 | 8 nt / 94.7 % | 10 | X – 308 – 280 – 280– 212 – 185 – 176 – 136 – 88 – 60 | | Human, Wound | | Bourg en Bresse, Fr, 2006 |
|  | ADV103 | 4+11 | 5 nt / 96.7% | 10 | X – 295 – 242 – 214– 163 – 145 – 145 – 100 – 57 – 41 | | Human, Stool | | Montpellier, Fr, 2008 |
|  | ADV109 | 4+11 | 5 nt / 96.7% | 10 | X – 307 – 254 – 220– 145 – 145 – 145 – 100 – 57 – 41 | | Human, Stool | | Montpellier, Fr, 2008 |
|  | ADV119 | 11 | - | 10 | X – 303 – 253 – 218– 166 – 152 – 152 – 133 – 61 – 44 | | Human, Stool | | Montpellier, Fr, 2009 |
|  | ADV125 | 11 | - | 10 | X – 306 – 262 – 220– 177 – 156 – 128 – 92 – 58 – 34 | | Human, Stool | | Montpellier, Fr, 2009 |
|  | ADV127 | 11 | - | 10 | X – 310 – 283 – 236– 194 – 180 – 111 – 89 – 58 – 32 | | Human, Stool | | Montpellier, Fr, 2009 |
|  | ADV130 | 11 | - | 10 | X – 318 – 252 – 217– 162 – 162 – 151 – 95 – 58 – 39 | | Human, Blood | | Montpellier, Fr, 2010 |
|  | ADV131 | 11 | - | 10 | X – 331 – 252 – 199– 172 – 162 – 162 – 117 – 68 – 44 | | Human, Respiratory tract | | Montpellier, Fr, 2009 |
|  | ADV135 | 11 | - | 10 | X – 311 – 311 – 220– 170 – 170 – 157 – 130 – 58 – 34 | | Human, Stool | | Montpellier, Fr, 2010 |
|  | ADV137b | 11 | - | 10 | X – 311 – 248 – 203– 157 – 151 – 130 – 92 – 58 – 24 | | Human, Respiratory tract | | Montpellier, Fr, 2010 |
|  | AK219 | 11 | - | 10 | X – 360 – 285 – 263– 194 – 180 – 180 – 128 – 88 – 65 | | Environment, Waste water treatment lagoon | | Montracol, Fr, 2006 |
|  | AK222 | 11 | - | 10 | X – 410 – 333 – 303– 253 – 198 – 180 – 128 – 88 – 65 | | Environment, Waste water treatment lagoon | | Montracol, Fr, 2006 |
|  | AK226 | 11 | - | 10 | X – 308 – 258 – 224– 181 – 164 – 164 – 102 – 68 – 40 | | Environment, Waste water treatment lagoon | | Montracol, Fr, 2006 |
|  | AK227 | 11 | - | 10 | X – 314 – 263 – 220– 172 – 159 – 141 – 111 – 60 – 40 | | Environment, Waste water treatment lagoon | | Montracol, Fr, 2006 |
|  | AK232 | 11 | - | 10 | X – 308 – 268 – 224– 181 – 172 – 150 – 92 – 60 – 40 | | Environment, Waste water treatment lagoon | | Montracol, Fr, 2006 |
|  | AK237 | 11 | - | 11 | X – 380 – 314 – 291– 258 – 224 – 185 – 172 – 102 – 68 – 40 | | Environment, Water lake | | Annecy, Fr, 1998 |
|  | AK238 | 11 | - | 10 | X – 303 – 279 – 224– 172 – 155 – 136 – 111 – 60 – 40 | | Environment, Water lake | | Annecy, Fr, 1998 |
|  | AK239 | 11 | - | 10 | X – 308 – 258 – 211– 181 – 159 – 159 – 97 – 68 – 40 | | Environment, Water lake | | Annecy, Fr, 1998 |
|  | AK240 | 11 | - | 10 | X – 322 – 269 – 242– 204 – 166 – 156 – 102 – 62 – 39 | | Environment, Water lake | | Annecy, Fr, 1998 |
|  | AK241 | 13 | - | 10 | X – 309 – 296 – 260– 188 – 161 – 161 – 124 – 73 – 44 | | Non-human, Snail | | Angers, Fr, 1995 |
|  | AK242 | 11 | - | 10 | X – 343 – 278 – 231– 205 – 161 – 161 – 118 – 73 – 39 | | Environment, Water lake | | Annecy, Fr, 1998 |
|  | AK243 | 3+11 | 6 nt / 96% | 10 | X – 322 – 272 – 231 – 188 – 161 – 161 – 118 – 67 – 43 | | Non-human, Snail | | Angers, Fr, 1995 |
|  | AK244 | 11 | - | 10 | X – 322 – 272 – 248 – 161 – 161 – 161 – 113 – 67 – 43 | | Environment, Water lake | | Annecy, Fr, 1998 |
|  | AK246 | 11 | - | 10 | X – 303 – 269 – 209 – 165 – 150 – 135 – 116 – 62 – 44 | | Non-human, Snail | | Angers, Fr, 1995 |
|  | AK247 | 11 | - | 10 | X – 309 – 258 – 237 – 165 – 150 – 140 – 116 – 62 – 44 | | Environment, Water lake | | Annecy, Fr, 1998 |
|  | AK248 | 11 | - | 10 | X – 303 – 258 – 220 – 170 – 160 – 135 – 116 – 70 – 39 | | Environment, Water lake | | Annecy, Fr, 1998 |
|  | **AK250** | 11 | - | 10 | X – 316 – 280 – 242 – 189 – 165 – 165 – 106 – 70 – 44 | | Environment, Water lake | | Annecy, Fr, 1998 |
|  | AK251 | 11 | - | 10 | X – 339 – 279 – 258 – 170 – 160 – 145 – 123 – 66 – 44 | | Environment, Water lake | | Annecy, Fr, 1998 |
|  | ***A. culicicola* CIP 107763^T^** | 4+11 | 5 nt / 96.7% | 10 | X – 305 – 263 – 215 – 167 – 151 – 151 – 107 – 58 – 29 | | Non-human, Mosquito midgut | | Pune, India, 1997 |
|  | *A. ichthiosmia* CECT 4486^T^ | 11 | - | 10 | X – 325 – 263 – 205 – 162 – 146 – 146 – 97 – 63 – 39 | | Environment, Surface water | | NA, Germany, 1986 |
|  | *A. veronii* bv. *sobria* LMG 13067 | 11 | - | 10 | X – 325 – 276 – 220 – 188 – 167 – 162 – 113 – 63 – 44 | | Non-human, Frog | | Connecticut, USA, NA |
|  | *A. veronii* CECT 4902 | 11 | - | 10 | X – 314 – 266 – 242 – 169 – 169 – 151 – 97 – 66 – 34 | | Environment, NA | | NA, Germany, 1993 |
|  | *A. veronii* CECT 7059 | 11 | - | 10 | X – 314 – 284 – 249 – 194 – 163 – 163 – 151 – 66 – 44 | | Environment, Drinking water | | Zaragoza, Spain, 2002 |
| ***A. hydrophila***  **(n=35)** | BVH3 | 1 | - | ND | ND | | Human, Wound | | Cahors, Fr, 2006 |
|  | BVH14 | 1+8 | 6 nt / 96% | 10 | X – 456 – 456 – 221 – 125 – 120 – 93 – 87 – 53 – 45 | | Human, Wound | | Le Mans, Fr, 2006 |
|  | *A. hydrophila* subsp. *hydrophila* CCM 2278 | 1+18 | 11 nt / 92.7% | 10 | X – 456 – 456 – 223 – 113 – 113 – 102 – 53 – 53 – 45 | | Non-human,  Red-legged frog | | California, USA, 1963 |
|  | BVH29 | 1+30 | 3 nt / 98% | 10 | X – 436 – 436 – 194 – 115 – 115 – 102 – 74 – 74 – 40 | | Human, Wound | | Reunion Island, Fr, 2006 |
|  | BVH30 | 1+30 | 3 nt / 98% | 10 | X – 436 – 436 – 194 – 115 – 115 – 102 – 74 – 74 – 40 | | Human, Stool | | Langres, Fr, 2006 |
|  | BVH35 | 1 | - | 10 | X – 479 – 479 – 248 – 163 – 131 – 131 – 80 – 80 – 44 | | Human, Wound | | La Roche sur Yon, Fr, 2006 |
|  | BVH99 | 1 | - | 10 | X – 502 – 464 – 237 – 120 – 120 – 120 – 75 – 75 – 44 | | Human, NA | | Brest, Fr, ND |
|  | BVH25a | 1+5 | 1 nt / 99.3% | 10 | X – 508 – 436 – 249 – 115 – 99 – 99 – 62 – 62 – 46 | | Human, Respiratory tract | | Saint-Brieux, Fr,2006 |
|  | BVH27b | 1+5 | 1 nt / 99.3% | 10 | X – 508 – 436 – 249 – 115 – 99 – 99 – 62 – 62 – 46 | | Human, Wound | | Reunion Island, Fr, 2006 |
|  | AK204 | 1+5 | 1 nt / 99.3% | 10 | X – 520 – 436 – 225 – 125 – 125 – 110 – 70 – 70 – 39 | | Non-human, Snail | | Angers, Fr, 1995 |
|  | BVH2 | 1 | - | 10 | X – 508 – 416 – 248 – 152 – 122 – 122 – 106 – 54 – 39 | | Human, Wound | | Cahors, Fr, 2006 |
|  | BVH12 | 1 | - | ND | ND | | Human, Eyes infection | | Cherbourg, Fr, 2006 |
|  | BVH24 | 1 | - | 10 | X – 436 – 436 – 194 – 115 – 115 – 97 – 62 – 62 – 39 | | Human, Wound | | Saint-Brieux, Fr,2006 |
|  | BVH33 | 1 | - | 10 | X – 479 – 479 – 235 – 131 – 131 – 131 – 86 – 75 – 44 | | Human, Wound | | La Rochelle, Fr, 2006 |
|  | BVH34 | 1+30 | 3 nt / 98% | 10 | X – 479 – 479 – 207 – 137 – 131 – 131 – 86 – 75 – 44 | | Human, Urine | | La Roche sur Yon, Fr, 2006 |
|  | BVH36 | 1 | - | 10 | X – 479 – 479 – 228 – 137 – 131 – 131 – 80 – 80 – 44 | | Human, Wound | | La Roche sur Yon, Fr, 2006 |
|  | BVH41 | 1+30 | 3 nt / 98% | 10 | X – 491 – 446 – 235 – 131 – 131 – 131 – 75 – 75 – 42 | | Human, Wound | | Vannes, Fr, 2006 |
|  | BVH42 | 1+35 | ND | 10 | X – 479 – 479 – 228 – 137 – 131 – 131 – 80 – 80 – 39 | | Human, Wound | | Périgueux, Fr, 2006 |
|  | BVH45 | 1 | - | 10 | X – 448 – 448 – 233 – 130 – 121 – 112 – 70 – 70 – 44 | | Human, Wound | | Périgueux, Fr, 2006 |
|  | BVH64 | 1+9+10 | 12 nt / 92% (max) | 10 | X – 448 – 448 – 200 – 121 – 112 – 112 – 70 – 70 – 44 | | Human, Wound | | Belfort, Fr, 2006 |
|  | BVH72 | 1 | - | 10 | X – 448 – 448 – 200 – 121 – 121 – 112 – 70 – 70 – 44 | | Human, Blood | | Martinique Island,Fr, ND |
|  | BVH75 | 1+39 | ND | 10 | X – 465 – 465 – 239 – 126 – 120 – 120 – 83 – 83 – 53 | | Human, Respiratory tract | | Saint-Etienne, Fr,2006 |
|  | BVH93 | 1 | - | 10 | X – 445 – 445 – 215 – 131 – 131 – 131 – 83 – 83 – 53 | | Human, Wound | | Cahors, Fr, 2006 |
|  | BVH96 | 1 | - | 10 | X – 445 – 445 – 226 – 120 – 120 – 120 – 79 – 79 – 49 | | Human, Wound | | Bourg en Bresse, Fr, 2006 |
|  | BVH97 | 1 | - | 10 | X – 452 – 452 – 226 – 130 – 130 – 130 – 73 – 73 – 44 | | Human, Wound | | Bourg en Bresse, Fr, 2006 |
|  | ADV105 | 1+2 | 1 nt / 99.3% | 10 | X – 468 – 405 – 217 – 113 – 113 – 97 – 97 – 63 – 36 | | Human, Stool | | Montpellier, Fr, 2008 |
|  | AK203 | 1 | - | 10 | X – 476 – 424 – 226 – 118 – 110 – 110 – 73 – 73 – 44 | | Non-human, Snail | | Angers, Fr, 1995 |
|  | AK218 | 1 | - | 10 | X – 447 – 416 – 203 – 127 – 122 – 122 – 75 – 65 – 40 | | Environment, Waste water treatment lagoon | | Montracol, Fr, 2006 |
|  | AK235 | 1+2 | 1 nt / 99.3% | 10 | X – 440 – 411 – 199 – 126 – 126 – 120 – 75 – 66 – 44 | | Environment, Waste water treatment lagoon | | Montracol, Fr, 2006 |
|  | *A. hydrophila* subsp. *hydrophila* CECT 839^T^ | 1 | - | 10 | X – 446 – 394 – 235– 120 – 120 – 98 – 70 – 70 – 39 | | Environment,Tin of milk with a fishy odor | | NA, NA, NA |
|  | *A. hydrophila* subsp. *ranae* CIP 107985 | 1+2 | 1 nt / 99.3% | 10 | X – 448 – 398 – 243 – 116 – 116 – 116 – 62 – 62 – 39 | | Non-human, Frog | | NA, Thaïland, NA |
|  | *A. hydrophila* CECT 5734 | 1 | - | 10 | X – 492 – 436 – 221 – 122 – 122 – 105 – 73 – 66 – 39 | | Non-human, Fish | | Valencia, Spain, 1987 |
|  | *A. hydrophila* subsp. *hydrophila* CCM 2280 | 1 | - | 10 | X – 448 – 448 – 194 – 118 – 118 – 118 – 67 – 67 – 39 | | Non-human, Snake | | NA, NA, 1963 |
|  | *A. hydrophila* subsp. *hydrophila* CCM 2282 | 1+4 | 12 nt / 92% | 10 | X – 448 – 448 – 235 – 121 – 121 – 103 – 73 – 73 – 39 | | Non-human, Nile Monitor | | NA, NA, 1963 |
|  | *A. hydrophila* subsp. *hydrophila* CCM 4528 | 1+10 | 12 nt / 92% | 10 | X – 448 – 448 – 200 – 130 – 130 – 115 – 73 – 66 – 44 | | Human, Stool | | NA, Czech Republic, 1993 |
| ***A. caviae***  **(n=34)** | BVH16 | 4 | - | 10 | X - 388 – 372 – 196 – 142 – 119 – 119 – 82 – 81 – 36 | | Human, Respiratory tract | | Rambouillet, Fr, 2006 |
|  | BVH57 | 3+4+40 | 2 nt / 98.7% (max) | 10 | X - 384 – 363 – 198 – 145 – 133 – 127 – 75 – 75 – 39 | | Human, Blood | | Versailles, Fr, 2006 |
|  | BVH63 | 1+4+18 | 12 nt / 92% (max) | 10 | X - 393 – 393 – 218 – 145 – 145 – 133 – 108 – 59 – 34 | | Human, Blood | | Macon, Fr, 2006 |
|  | BVH84 | 1+4+18 | 12 nt / 92% (max) | 10 | X - 382 – 382 – 191 – 149 – 142 – 142 – 102 – 69 – 42 | | Human, Stool | | Aix en Provence, Fr, 2006 |
|  | BVH98 | 3+4 | 1 nt / 99.3% | 10 | X - 408 – 384 – 200 – 158 – 145 – 115 – 115 – 59 – 34 | | Human, Wound | | Brest, Fr, NA |
|  | ADV118 | 1+4+8 | 12 nt / 92% (max) | 10 | X - 377 – 367 – 215 – 133 – 133 – 109 – 103 – 62 – 34 | | Human, Wound | | Montpellier, Fr, 2009 |
|  | ADV121 | 1+3+4 | 12 nt / 92% (max) | 10 | X - 348 – 348 – 201 – 146 – 133 – 118 – 109 – 61 – 39 | | Human, Stool | | Montpellier, Fr, 2009 |
|  | BVH48 | 3+4 | 1 nt / 99.3% | 10 | X - 403 – 394 – 196 – 151 – 144 – 127 – 74 – 74 – 40 | | Human, Vagina | | Monceau les mines, Fr, 2006 |
|  | *A. caviae* CCUG 48892 | 3+4+18 | 2 nt / 98.7% (max) | 10 | X - 378 – 365 – 224 – 145 – 133 – 127 – 109 – 68 – 36 | | Environment,  Water | | Uppsala, Sweden, 2004 |
|  | BVH19 | 3+4+11 | 6 nt / 96% (max) | 10 | X - 403 – 380 – 196 – 151 – 144 – 133 – 74 – 74 – 40 | | Human, Vagina | | Villeneuve sur Lot, Fr, 2006 |
|  | BVH81 | 3+4+11+15 | 13 nt / 91.3% (max) | 10 | X - 408 – 384 – 200 – 173 – 145 – 124 – 65 – 65 – 34 | | Human, Stool | | Aix en Provence, Fr, 2006 |
|  | BVH66 | 4+18 | 1 nt / 99.3% | 10 | X - 466 – 404 – 237 – 145 – 115 – 115 – 65 – 65 – 34 | | Human, Wound | | Martinique Island, Fr, 2006 |
|  | BVH55 | 3+4+11+18 | 6 nt / 96% (max) | 10 | X - 417 – 388 – 196 – 162 – 146 – 129 – 119 – 75 – 48 | | Human, Stool | | Saint-Denis, Fr, 2006 |
|  | BVH87 | 3+4+15 | 13 nt / 91.3% (max) | 10 | X - 384 – 384 – 200 – 145 – 115 – 115 – 65 – 65 – 34 | | Human, Stool | | Aix en Provence, Fr, 2006 |
|  | BVH4 | 3+4+11 | 6 nt / 96% (max) | 11 | X - 443 – 390 – 360 – 308 – 186 – 168 – 134 – 118 – 69 - 42 | | Human, Wound | | Cahors, Fr, 2006 |
|  | BVH15 | 4 | - | 10 | X - 391 – 371 – 198 – 143 – 109 – 109 – 58 – 58 – 26 | | Human, Blood | | Grasse, Fr, 2006 |
|  | BVH20 | 3+4+6+15 | 13 nt / 91.3% (max) | 10 | X - 380 – 371 – 221 – 158 – 120 – 120 – 74 – 74 – 40 | | Human, Stool | | Gonesse, Fr, 2006 |
|  | BVH51 | 4 | - | 10 | X - 403 – 380 – 196 – 151 – 144 – 127 – 74 – 74 – 40 | | Human, Blood | | Monaco, Fr, 2006 |
|  | BVH52 | 4 | - | 10 | X - 382 – 360 – 191 – 142 – 129 – 110 – 69 – 69 - 42 | | Human, Blood | | Monaco, Fr, 2006 |
|  | BVH67 | 3+4+12 | 2 nt / 98.7% (max) | 10 | X - 393 – 393 – 200 – 145 – 124 – 115 – 115 – 65 – 34 | | Human, Stool | | Martinique Island, Fr, NA |
|  | BVH85 | 3+4 | 1 nt / 99.3% | 10 | X - 381 – 381 – 238 – 159 – 132 – 132 – 85 – 85 – 57 | | Human, Stool | | Aix en Provence, Fr, 2006 |
|  | BVH86 | 1+4 | 12 nt / 92% | 10 | X - 393 – 393 – 198 – 145 – 115 – 115 – 65 – 65 – 34 | | Human, Stool | | Aix en Provence, Fr, 2006 |
|  | BVH100 | 3+4 | 1 nt / 99.3% | 10 | X - 455 – 408 – 280 – 163 – 158 – 124 – 108 – 65 – 34 | | Human, Wound | | Brest, Fr, ND |
|  | ADV106 | 4 | - | 10 | X - 380 – 380 – 184 – 148 – 133 – 106 – 57 – 57 – 27 | | Human, Stool | | Montpellier, Fr, 2008 |
|  | ADV124 | 4 | - | 10 | X - 365 – 365 – 188 – 140 – 123 – 111 – 64 – 64 – 34 | | Human, Stool | | Montpellier, Fr, 2009 |
|  | AK223 | 4 | - | 10 | X - 403 – 369 – 205 – 144 – 129 – 129 – 69 – 69 – 44 | | Environment, Waste water treatment lagoon | | Montracol, Fr, 2006 |
|  | AK229 | 4+18+19 | 2 nt / 98.7% (max) | 10 | X - 507 – 285 – 205 – 144 – 134 – 110 – 69 – 69 – 39 | | Environment, Waste water treatment lagoon | | Montracol, Fr, 2006 |
|  | AK231 | 3+4 | 1 nt / 99.3% | 10 | X - 443 – 413 – 205 – 149 – 144 – 129 – 75 – 75 – 44 | | Environment, Waste water treatment lagoon | | Montracol, Fr, 2006 |
|  | AK234 | 3+4+18 | 2 nt / 98.7% (max) | 10 | X - 472 – 382 – 215 – 142 – 134 – 134 – 110 – 75 – 44 | | Environment, Waste water treatment lagoon | | Montracol, Fr, 2006 |
|  | AK245 | 3+4+11+12 | 6 nt / 96% (max) | 10 | X - 404 – 365 – 198 – 145 – 128 – 111 – 62 – 62 – 34 | | Environment, Water lake | | Annecy, Fr, 1998 |
|  | ***A. caviae* CECT 838^T^** | 4+18 | 1 nt / 99.3% | 10 | X - 411 – 380 – 215 – 129 – 102 – 102 – 58 – 58 – 24 | | Non-human, Guinea pig | | NA, USA, NA |
|  | *A. hydrophila* subsp. *anaerogenes* CECT 4221 | 3+4 | 1 nt / 99.3% | 11 | X - 411 – 395 – 371 – 243 – 220 – 173 – 146 – 146 – 73 – 26 | | Environment, Used oil emulsion | | NA, USA, NA |
|  | *A. caviae* CECT 4222 | 4 | - | 10 | X - 381 – 381 – 186 – 130 – 130 – 108 – 56 – 56 – 34 | | Environment, Sewage | | NA, NA, 1954 |
|  | *A. caviae* CECT 4226 | 3+4 | 1 nt / 99.3% | 10 | X - 428 – 369 – 202 – 146 – 146 – 108 – 108 – 64 – 34 | | Environment, Used oil emulsion | | NA, USA, 1953 |
| ***A. piscicola***  **(n=3)** | *A. piscicola* LMG 24783^T^ | 30 | - | 10 | X - 457 – 415 – 194 – 164 – 115 – 106 – 106 – 63 – 51 | | Non-human, Salmon | | Gallicia, Spain, 2005 |
|  | *A. sobria* CECT 4333 | 30 | - | 10 | X - 444 – 444 – 226 – 151 – 123 – 123 – 71 – 71 -39 | | Non-human, Diseased elver | | Valencia, Spain, NA |
|  | *Aeromonas* sp. CECT 5177 | 30 | - | 10 | X - 442 – 442 – 221 – 152 – 121 – 121 – 73 – 67 -39 | | Environment, Drinking water | | Eeklo, Belgium, 1996 |
| ***A. salmonicida***  **(n=8)** | *A. salmonicida* subsp. *achromogenes* CIP 104001 | 30 | - | 9 | X - 545 – 398 – 197 – 172 – 172 – 114 – 67 – 35 | Non-human, Trout | | Aberdeen, UK, 1963 | |
|  | *A. salmonicida* subsp. *masoucida* CIP 103210 | 30 | - | 9 | X - 582 – 410 – 218 – 172 – 131 – 108 – 70 – 35 | Non-human, Fish blood | | NA, NA, 1969 | |
|  | *A. salmonicida* subsp. *smithia* CIP 104757 | 30 | - | 9 | X - 582 – 410 – 218 – 172 – 131 – 108 – 70 – 35 | Non-human, Fish ulcer | | NA, UK, NA | |
|  | *A. salmonicida* subsp. *salmonicida* CIP 103209^T^ | 30 | - | 9 | X - 485 – 457 – 218 – 172 – 160 – 108 – 67 – 35 | Non-human, Diseased salmon | | Cletter river, UK, 1953 | |
|  | **BVH39** | 30 | - | 10 | X - 448 – 340 – 231 – 188 – 129 – 107 – 70 – 65 – 35 | Human, Wound | | Vannes, Fr, 2006 | |
|  | *A. salmonicida* subsp. *pectinolytica* CIP 107036 | 30 | - | 10 | X - 431 – 348 – 212 – 212 – 172 – 114 – 86 – 67 -35 | Environment,  River water | | Buenos Aires, Argentina, NA | |
|  | *A. salmonicida* CCM 1150 | 30 | - | 10 | X - 495 – 345 – 230 – 167 – 119 – 108 – 67 – 67 - 35 | Non-human,  Fish | | NA, Czech Republic, 1961 | |
|  | *A. salmonicida* CCM 1275 | 30 | - | 10 | X - 460 – 367 – 214 – 169 – 169 – 127 – 91 – 79 - 44 | Non-human,  Fish | | NA, Czech Republic, 1961 | |
| ***A. allosaccharophila***  **(n=3)** | BVH88 | 13 | - | 10 | X – 348 – 262 – 210 – 170 – 157 – 157 – 107 – 65 – 34 | Human, Blood | | Dunkerque, Fr, 2006 | |
|  | *A. allosaccharophila* CECT 4199^T^ | 13 | - | 10 | X – 291 – 220 – 178 – 134 – 134 – 118 – 97 – 58 – 44 | Non-human, Fish | | Valencia, Spain, 1991 | |
|  | *A. sobria* CECT 4053 | 13 | - | 10 | X – 308 – 234 – 217 – 161 – 146 – 130 – 108 – 56 – 49 | Environment,  Activated sludge | | Stockholm, Sweden, 1978 | |
| ***A. sobria***  **(n=5)** | *A. sobria* CECT 4245^T^ | 38 | - | 10 | X – 306 – 256 – 242 – 154 – 154 – 143 – 77 – 53 – 44 | Non-human, Fish | | NA, Fr, 1974 | |
|  | *Aeromonas* sp. CECT 4816 | 38 | - | 10 | X – 308 – 274 – 225 – 169 – 169 – 146 – 108 – 71 – 49 | Non-human, Fish | | NA, NA, 1993 | |
|  | *Aeromonas* sp. CECT 4817 | 38 | - | 10 | X – 345 – 263 – 243 – 169 – 169 – 153 – 100 – 71 – 49 | Non-human, Fish | | NA, NA, 1993 | |
|  | *Aeromonas* sp. CECT 4818 | 38 | - | ND | ND | Non-human, Fish | | NA, NA, 1993 | |
|  | *A. sobria* CECT 4821 | 38 | - | 10 | X – 314 – 263 – 221 – 178 – 169 – 163 – 102 – 66 – 39 | Non-human, Fish | | NA, NA, 1993 | |
| ***A. aquariorum***  **(n=8)** | BVH28b | 3 | - | 10 | X – 436 – 436 – 249 – 124 – 100 – 100 – 65 – 65 – 46 | Human, Wound | | Reunion Island, Fr, 2006 | |
|  | BVH43 | 3 | - | 9 | X – 473 – 448 – 254 – 130 – 112 – 112 – 70 – 44 | Human, Wound | | Périgueux, Fr, 2006 | |
|  | BVH65 | 3 | - | 9 | X -473 – 448 – 200 – 121 – 112 – 112 – 70 – 44 | Human, Blood | | Martinique Island, Fr, 2006 | |
|  | BVH68 | 3+35 | ND | 10 | X - 481 – 443 – 281 – 125 – 106 – 106 – 73 – 73 – 38 | Human, NA | | Martinique Island, Fr, ND | |
|  | BVH70 | 3+4 | 1 nt / 99.3% | 10 | X - 449 – 449 – 275 – 123 – 123 – 109 – 75 – 75 - 44 | Human, NA | | Martinique Island, Fr, ND | |
|  | ADV132 | 2+3 | 10 nt / 93.4% | 10 | X - 453 – 403 – 242 – 111 – 111 – 111 – 64 – 64 - 44 | Human, Wound | | Montpellier, Fr, 2010 | |
|  | *A. hydrophila* subsp. *dhakensis* CIP 107500 | 3 | - | 10 | X – 444 -444 – 240 – 133 – 104 – 104 – 67 – 67 – 37 | Human, Stool | | NA, Bangladesh, NA | |
|  | *A. aquariorum* CECT 7289^T^ | 3 | - | 10 | X – 448 – 394 – 235 – 109 – 109 – 109 – 62 – 62 – 30 | Non-human, Fish | | NA, Portugal, 2003 | |
| ***A. media***  **(n=6)** | BVH40 | 11+20 | 6 nt / 96% | 10 | X – 485 – 277 – 236 – 122 – 122 – 103 – 54 – 54 – 32 | Human, Stool | | Vannes, Fr, 2006 | |
|  | AK202 | 21 | - | 10 | X – 426 – 349 – 200 – 122 – 122 – 103 – 54 – 37 – 37 | Non-human, Snail | | Angers, Fr, 1995 | |
|  | AK211 | 15+20+21 | 9 nt / 94% (max) | 10 | X – 358 – 331 – 203 – 182 – 139 – 109 – 109 – 60 – 34 | Non-human, Snail | | Angers, Fr, 1995 | |
|  | ***A. media* CECT 4232^T^** | 15 | - | 10 | X – 339 - 339 – 226 – 140 – 124 – 107 – 107 – 58 – 24 | Environment, Fish farm effluent water | | NA, UK, NA | |
|  | *Aeromonas* sp. CECT 7111 | 2+15+21 | 9 nt / 94% (15+21) | 10 | X – 345 – 296 – 194 – 138 – 119 – 108 – 90 – 49 – 27 | Non-human, Oyster | | Barcelona, Spain, NA | |
|  | ***A. media* CCM 4242** | 20+21 | 7 nt / 95.4% | 10 | X – 388 – 388 – 201 – 140 – 140 – 121 – 121 – 76 – 44 | Environment, River water | | NA, Czech Republic, 1991 | |
| ***A. tecta***  **(n=3)** | *A. tecta* CECT 7082^T^ | 15 | - | 10 | X - 456 – 354 – 157 – 145 – 127 – 97 – 73 – 54 – 34 | Human, Stool | | Ticino, Switzerland, NA | |
|  | *Aeromonas* sp. CECT 7081 | 15 | - | 10 | X - 424 – 388 – 221 – 145 – 127 – 127 – 97 -72 - 44 | Non-human, Fish | | Ticino, Switzerland, 1983 | |
|  | *Aeromonas* sp. CECT 7083 | 15 | - | 10 | X - 451 – 357 – 153 – 138 – 119 – 90 – 49 – 49 -27 | Environment, Tap water | | Ticino, Switzerland, 1993 | |
| ***A. jandaei***  **(n=2)** | BVH92 | 4 | - | 10 | X – 315 – 276 – 215 – 194 – 170 – 157 – 92 – 65 – 34 | Human, Urine | | Toulouse, Fr, 2006 | |
|  | *A. jandaei* CECT 4228^T^ | 4 | - | 10 | X – 309 – 258 – 207 – 188 – 158 – 158 – 97 – 63 – 39 | Human, Stool | | Oregon, USA, 1980 | |
| ***A. enteropelogenes*** | ***A. enteropelogenes* CECT 4487^T^** | 3+4+36 | 2 nt / 98.7% (max) | 10 | X – 287 – 260 – 222 – 174 – 133 – 133 – 88 – 59 – 39 | Human, Stool | | NA, India, NA | |
| ***A. trota*** | ***A. trota* CECT 4255^T^** | 4+15 | 12 nt / 92% | 10 | X – 287 – 260 – 222 – 178 – 133 – 133 – 92 – 61 – 39 | Human, Stool | | Varasani, India, NA | |
| ***A. bestiarum*** | *A. bestiarum* CECT 4227^T^ | 30 | - | 9 | X - 448 – 400 – 228 – 118 – 118 – 63 – 63 – 39 | Non-human, Fish | | NA, Fr, 1974 | |
| ***A. encheleia*** | *A. encheleia* CECT 4342^T^ | 1+30 | 3 nt / 98% | 10 | X - 400 – 363 – 194 – 111 – 111 – 97 – 69 – 56 – 34 | Non-human, Fish | | Valencia, Spain, 1987 | |
| **HG11** | HG11 CECT 4253^T^ | 15+30 | 5 nt / 96.7% | 10 | X - 339 – 314 – 175 – 145 – 133 – 115 – 115 – 61 – 34 | Human, Wound | | New Zealand, 1983 | |
| ***A. eucrenophila*** | *A. eucrenophila* CECT 4224^T^ | 15 | - | 10 | X - 437 – 388 – 194 – 139 – 118 – 104 – 63 – 63 – 34 | Non-human, Freshwater fish | | NA, NA, NA | |
| ***A. fluvialis*** | ***A. fluvialis* LMG 24681^T^** | 14+30 | 1 nt / 99.3% | 9 | X - 270 – 236 – 182 – 176 – 170 – 97 – 79 – 40 | Environmental, River water | | Girona, Spain, NA | |
| ***A. popoffii*** | *A. popoffi* CIP 105493^T^ | 15+29 | 5 nt / 96.7% | 10 | X - 410 – 410 – 200 – 114 – 114 – 86 – 59 – 35 – 35 | Environmental, Water | | Oelegem, Belgium, 1993 | |
| ***A. sanarellii*** | *A. sanarellii* LMG 24682^T^ | 3+18 | 2 nt / 98.7% | 10 | X - 408 – 224 – 212 – 133 – 105 – 105 – 63 – 57 – 32 | Human, Wound | | NA, Taïwan, 2000 | |
| ***A. schubertii*** | *A. schubertii* CECT 4240^T^ | 37 | - | 8 | X - 582 – 230 – 200 – 174 – 137 – 35 – 27 | Human, Wound | | Texas, USA, 1981 | |
| ***A.* *diversa*** | HG13 CECT 4254^T^ | 11 | - | 8 | X - 566 – 201 – 157 – 157 – 73 – 36 – 30 | Human, Wound | | Louisiana, USA, NA | |
| ***A. taiwanensis*** | *A. taiwanensis* LMG 24683^T^ | 3+31 | 10 nt / 93.4% | 10 | X - 370 – 356 – 197 – 127 – 103 – 103 – 60 – 51 – 27 | Human, Wound | | NA, Taïwan, 2000 | |
| **Unknown taxon** | ***A. bestiarum* CCM 1271** | 30 | - | 10 | X - 426 – 416 – 221 – 102 – 102 – 92 – 49 – 49 – 24 | Non-human, Gold fish | | NA, NA, NA | |
| ***A. bivalvium*** | *A. bivalvium* CECT 7113^T^ | 27 | - | 10 | X - 412 – 351 – 164 – 158 – 158 – 146 – 63 – 63 – 34 | Non-human, Cockles | | Barcelona, Spain, 1997 | |
| ***A. molluscorum*** | *A. molluscorum* CIP 108876^T^ | 1+2+24+25 | 2 nt / 98.7% (1+2+24 max) | 10 | X - 448 – 363 – 188 – 158 – 139 – 139 – 97 – 63 – 39 | Non-human, Wedge-shells | | Barcelona, Spain, 1997 | |
| ***A. simiae*** | *A. simiae* CIP 107798^T^ | 11 | - | 8 | X - 517 – 350 – 145 – 145 – 97 – 30 – 30 | Non-human, Healthy monkey | | NA, Mauritus, 1999 | |
| ***A. rivuli*** | *A. rivuli* DSM 22539^T^ | 32 | - | 10 | X - 400 – 318 – 157 – 133 – 133 – 133 – 94 – 75 – 32 | Environment, Karst hardwater creek | | Westerhöfer Bach, Germany, NA | |

^a^ according to Roger *et al*. [13].

^b^ PCR-TTGE patterns are designed by the combination of PCR-TTGE band numbers separated by a + sign.

^c^ *rrs* V3 region heterogeneity level indicated by the number of divergent nucleotides (nt) between sequences of bands in the PCR-TTGE patterns followed by the similarity level between band sequences (determined from a 151-bp alignment) . When sequences were not available for all bands in a pattern, values were given for the combination of sequenced bands among the profile given in parentheses. For patterns composed of 3 or 4 sequences bands, values were given for the more divergent copies within the PCR-TTGE pattern and this is indicated by max in parentheses.

^d^ Fragment with higher molecular weight could not be sized here as discussed in the text. When double or triple bands were noted on the basis of fragment intensity, they have been assigned an identical size.

^e^ All bacteremia originated from the gut [55].

MLPA: multilocus phylogenetic analysis; ND: not determined; NA: not available; -: not applicable.
